# Optimising parental self-efficacy in the neonatal intensive care unit (NICU) and its implications on child care and parenting quality: a study protocol

**DOI:** 10.1136/bmjopen-2026-116985

**Published:** 2026-05-25

**Authors:** Tina C Montreuil, Chloé Gratton, Kimia Mir-Shokraei, Valérie Biran, Marc Beltempo, Marie Brossard-Racine, Marion Lecorguillé

**Affiliations:** 1 Department of Educational and Counselling Psychology, McGill University Faculty of Education, Montreal, Quebec, Canada; 2 The Institute, McGill University Health Centre, Montreal, Quebec, Canada; 3 Department of Psychiatry, McGill University Faculty of Medicine and Health Sciences, Montreal, Quebec, Canada; 4 Department of Pediatrics, McGill University Faculty of Medicine and Health Sciences, Montreal, Quebec, Canada; 5 Centre de recherche Azrieli - CHU Sainte-Justine, Montreal, Quebec, Canada; 6 Department of Health Informatics, University of Waterloo, Waterloo, Ontario, Canada; 7 Neonatal Care and Neonatal Resuscitation Service, Hospital Robert Debré, Paris, France; 8 Department of Pediatrics, Division of Neonatology, McGill University Health Centre, Montreal, Quebec, Canada; 9 McGill University School of Physical & Occupational Therapy, Montreal, Quebec, Canada

**Keywords:** Neonatal intensive & critical care, Parents, Health, MENTAL HEALTH

## Abstract

**Introduction:**

Parenting an infant in the neonatal intensive care unit (NICU) can be highly stressful, undermine parental confidence and minimise involvement in infant care. Enhancing parental self-efficacy is therefore crucial to promoting active engagement and supporting positive child outcomes. This study aims to identify the psychosocial needs (emotion regulation and stress management) of NICU parents and examine the factors that influence their engagement in infant care. It also seeks to determine whether higher levels of parental self-efficacy are associated with greater engagement and collaboration with healthcare providers (HCPs) and a shorter NICU stay.

**Methods and analysis:**

A mixed-methods study will be conducted at the Montreal Children’s Hospital’s Level III NICU located in Quebec, Canada. Parents (ie, mothers and, when available, their partners) will be recruited during their infant’s hospitalisation. Participants will complete a set of self-report questionnaires measuring parental engagement, self-efficacy, parent-infant attachment, mental health, perceived stress and social support. Additionally, one parent per family will be invited to participate in a qualitative interview to discuss their experience in the NICU. Qualitative data will be analysed thematically using NVivo 12 to identify key themes, and statistical analyses will be conducted using RStudio to determine which factors are most strongly associated with parental engagement and their interactions with the clinical team. Finally, HCPs will be invited to assist and participate in a qualitative interview regarding their perspectives on the NICU environment and the study’s impact on the unit (ie, reduction in HCP burden due to increased parental engagement). Findings from this study will offer valuable insights to inform future clinical strategies aimed at enhancing parental engagement and self-efficacy in the NICU. These outcomes may contribute to improved neonatal outcomes, parental mental health and the advancement of evidence-based, family-centred care policies.

**Ethics and dissemination:**

The protocol was approved by the Montreal University Health Centre’s (MUHC) Research Ethics Board (2025-9392). Findings derived from this study have the potential to optimise parental engagement in the NICU to promote family resilience and child well-being.

**Implications of findings:**

Our study protocol aims to underscore the critical role of self-efficacy and other key psychosocial factors, such as lower parental stress and higher emotion regulation, in promoting parental engagement and collaboration within the NICU. We anticipate that higher levels of self-efficacy will correlate with parental well-being, increased caregiving engagement (ie, basic baby care, improved communication with NICU staff) and enhanced parent-infant bonding. These findings may support the development of structured interventions that empower parents and streamline provider-parent collaboration. In clinical practice, these insights can guide the integration of tailored parental support programmes to optimise discharge preparation and reduce provider burden by promoting shared caregiving responsibilities. Future initiatives could build further on this model to guide institutional policies that aim to prioritise parental empowerment (ie, greater self-efficacy and engagement) as a fundamental pillar of neonatal care.

**STRENGTHS AND LIMITATIONS OF THIS STUDY**

- Mixed-methods design combining quantitative and qualitative approaches to provide comprehensive insights into the psychosocial needs, caregiving experiences and perceived barriers faced by neonatal intensive care unit parents.
- Use of validated instruments to assess parental engagement, self-efficacy and mental health.
- Inclusion of both mothers and partners to capture dyadic experiences.
- Single-centre study may limit generalisability beyond the pilot implementation institution.
- No post-discharge follow-up limits the understanding of long-term impacts on parents’ and children’s outcomes.

## Introduction

Early life experiences play a pivotal role in shaping neurodevelopmental trajectories, particularly when adversity is faced during the critical periods of brain maturation.^1^ Challenges occurring during sensitive windows of brain development—such as premature exposure to the extra-uterine environment—have been linked to long-term disruptions in cognitive, social-emotional and neural functioning.^2^

Admission of an infant to the neonatal intensive care unit (NICU) is inherently stressful and often elicits acute psychological distress in most parents, which may evolve into post-traumatic stress disorders, anxiety and depression.^3^ This distress is intensified by NICU admission, typically prompted by acute or severe illness, where parents must also cope with the profound fear of losing their child. Parents tend to rely on certain coping styles to avoid becoming overwhelmed by perceived or actual threats to their child (ie, impairments or death), compounded by their feelings of own competence as a parent.^4^ Up to 35% of mothers and 24% of fathers report developing acute stress symptoms shortly after admission and, for many cases, persist well beyond discharge.^5–7^ Pre-existing maternal mental health conditions, such as anxiety or depression during pregnancy, may further increase the likelihood of a NICU admission and exacerbate the risk of postpartum mental illness.^8–11^ The NICU context, characterised by physical separation, complex medical technology and dependence on healthcare providers’ (HCPs) support, can be disruptive to the natural bonding and attachment processes. Consequently, many parents are left feeling unprepared and undermining their sense of competence.^12–14^

### Promoting parental well-being in the NICU

HCPs increasingly recognise the urgent need to address and support parental mental health in the NICU, while also fostering active parental engagement and attachment with the infant throughout hospitalisation and beyond.^13^ Parental engagement in the NICU refers to the active involvement of parents in their infant’s direct caregiving activities (ie, feeding, diapering, skin-to-skin, etc), emotional bonding (ie, eye contact and soothing) and collaborative decision-making with the NICU staff about treatment and discharge planning.^15 16^ Engagement extends beyond physical presence during hospitalisation; it reflects the degree to which parents participate in caregiving, communication and collaboration with the healthcare team in the NICU. Higher engagement has been shown to bridge gaps across the multiple providers and services that infants often require and underpin successful transitions to home while reducing preventable readmissions.^17 18^ However, emotional distress, the short length of NICU stays and competing demands are limiting factors for meaningful engagement.^13^ Understanding the emotional and psychological factors that promote or limit engagement are therefore essential to better support families during their transition from hospital to home.^19 20^

Within this framework, parental well-being can be promoted by targeting two key and interrelated processes: self-efficacy and emotion regulation. In the NICU context, parental sense of competence or self-efficacy refers to a parent’s belief in their ability to successfully carry out caregiving tasks and effectively respond to their infant’s needs, despite the stress and challenges of the intensive care environment.^21 22^ It is rooted in Bandura’s self-efficacy theory and is a key psychological resource for parents of preterm or medically fragile infants.^23^ Strengthening parental self-efficacy early in the NICU has been shown to foster more meaningful engagement, reduce parental distress and support healthier developmental outcomes.^24^ In parallel, emotion regulation, defined as the ability to monitor, recognise and regulate one’s emotional responses to stressful events, is equally critical for parents in the NICU environment.^25–27^ Unlike self-efficacy, emotion regulation relates to the parents’ ability to manage distress and reframe negative thoughts while remaining responsive to their infant’s cues among ongoing stressors.^28^ This regulatory capacity allows for parents to stay present and aware during medical decision making, take part in nurturing interactions even when experiencing fear or grief and avoid disengagement or withdrawal that can arise from overwhelming anxiety or traumatic stress.^28 29^ Higher levels of emotion regulation not only preserve parental mental health but also strengthen the consistency and quality of parent-infant bonding.^24 27^

Despite the recognised importance of engagement, parents continue to encounter accessibility and systemic challenges such as restrictive visiting hours, lack of on-site accommodation, challenges balancing work or caring for other children and emotional distress; all of which might constrain their ability to fully participate in NICU caregiving.^30–32^ Structured evidence-based early intervention programmes for parental support remain inconsistently implemented, limiting many families’ access to aid and resources to navigate their time in the NICU.^30 33–37^ Moreover, most psychosocial interventions in the NICU remain disproportionately focused on mothers.^3^ Fathers, although equally vulnerable to distress, are often underrepresented in studies and underserved in practice.^38 39^ Emotional and psychological support must be actively integrated into NICU care to ensure that all parents are equipped to engage meaningfully in their infant’s care, particularly those who remain underserved in both research and clinical practice.^13 40 41^ Importantly, early interventions are consistent with growing evidence indicating that preventative approaches are both more effective and more cost-efficient than post-discharge care.^42 43^ Given the multifaceted challenges that parents must face during NICU hospitalisation, it is beneficial to examine how the structure and delivery of care, including the role of HCPs, may contribute to mitigating these stressors.

### Care provider role in supporting parental well-being

The NICU environment can be overwhelming and stressful for most parents, including those who do not typically exhibit emotional fragility or psychological distress under ordinary circumstances. Characterised by atypical lighting, medical emergency-related noises and frequent medical procedure disruptions, the NICU environment presents daily challenges that may compromise parental well-being and, consequently, parent–infant bonding.^3^ The physical immaturity of preterm infants, often following difficult, unplanned or traumatic deliveries, may heighten parental distress and contribute to uncertainty about their baby’s chances of survival. These stressors are compounded by factors beyond the hospital such as restricted visitation, caregiving demands at home and emotional exhaustion. For new parents with pre-existing adverse childhood experiences or mental health conditions, recognising these challenges is particularly critical as it can enable HCPs to implement timely trauma-informed interventions that may mitigate the negative impacts of NICU-related stress and support parental well-being.^44^

HCPs play a pivotal role in alleviating or aggravating these challenges.^45^ Empathic communication, hands-on training and emotionally intelligent care have been identified as key factors that enhance parental confidence and engagement.^46^ However, care quality often varies due to differences in HCP training, workload and emotional capacity.^47^ Increasingly, COVID-19 has greatly contributed to heightened levels of burnout and compassion fatigue among providers,^48^ thus negatively affecting the quality of patient care in the post-COVID era. Relatedly, given the current demands placed on HCPs in the post-COVID context, fostering greater parental self-efficacy in NICU care may also help alleviate the burden of the NICU nursing staff. As such, critically assessing the lived experiences of HCPs, beyond those of knowledge users and parents, is of utmost importance to ensure the delivery of quality care during the challenging transition to parenthood.

Despite known benefits of HCP support on patient well-being, little is understood about the specific psychosocial and emotional needs of parents during NICU admission, and the factors that either promote or hinder their engagement.^49 50^ Additionally, gaps remain in understanding how parental engagement and self-efficacy relate to measurable outcomes, such as length of NICU stay and discharge readiness.

### Research objectives

The overarching goal of this study protocol is to improve our understanding of how to optimise parental self-efficacy and engagement in the NICU, and to inform interventions that could enhance childcare and parenting quality after NICU discharge. To do such, there are four specific aims:

1. Identify the psychosocial and emotional needs of parents in the NICU.
2. Evaluate factors that promote self-efficacy, parental engagement (ie, communication and collaboration with providers in neonatal care), and improve parental well-being in the NICU.
3. Identify barriers to parental engagement.
4. Evaluate the role of HCP collaboration in supporting parent self-efficacy and engagement with infant care.

Based on these aims and the literature, the hypotheses are the following:

It is hypothesised that parents presenting higher emotion regulation skills will demonstrate greater parental engagement in childcare during their stay in the NICU (Hypothesis 1). Second, we hypothesise that parents demonstrating greater engagement in childcare during their stay in the NICU will show increased participation and collaboration with HCPs in neonatal care (Hypothesis 2). Third, it is hypothesised that families exhibiting greater parental engagement will have children with shorter NICU stays and an earlier discharge, when accounting for medical conditions, in comparison to families who display poorer parental engagement (Hypothesis 3). Finally, it is hypothesised that increased levels of collaboration and support from HCPs will be significantly associated with greater parental self-efficacy and engagement during the NICU stay (Hypothesis 4).

## Methods and analysis

### Study design

This research is designed as an exploratory mixed-methods study combining quantitative online surveys and qualitative interviews. This approach aims to capture a comprehensive understanding of parental self-efficacy, engagement in NICU care and the contextual factors shaping parent–provider interactions.

The study will take place in the NICU of the Montreal Children’s Hospital (Quebec, Canada). Canadian Level III NICU units provide regional intensive care.^51^ NICU parent participants will include mothers of hospitalised infants and their partners (if applicable). NICU HCPs from the same institution will be recruited, including neonatologists, NICU nurses, social workers, psychologists, lactation consultants and other providers directly involved in neonatal care.

### Participant recruitment

#### Inclusion and exclusion criteria

Eligible parents must be 18 years or older, have an infant currently admitted to the Montreal Children’s Hospital’s NICU, hold a valid Régie de l'assurance maladie du Québec (RAMQ) number and provide informed consent in either English or French. Parents will be excluded if they do not meet the above-mentioned inclusion criteria or if their child is actively receiving end-of-life care, to avoid any additional emotional burden. HCPs must be 18 years or older, currently employed or affiliated with the Montreal Children’s Hospital’s NICU and can provide informed consent in either English or French. Providers who do not meet the inclusion criteria will be excluded.

#### Recruitment procedures

Parents (n=50) will be recruited through two strategies: (1) in-unit advertising posters and handouts with a QR code that links to a pre-screening survey and (2) in-person recruitment via the Montreal Children’s Hospital’s NICU Clinic Coordinator, NICU staff referrals and trained members of the study’s research team. Eligible parents who have scanned the QR code will receive the electronic-informed consent form (ie, e-consent) and a set of self-report questionnaires via email.

HCPs (n=15) will be recruited through two strategies: (1) email outreach and (2) in-person recruitment. The research team will email the Montreal Children’s Hospital’s NICU staff with an overview of the study and a participation link. Interested providers can access the e-consent and enrolment form directly through this email.

#### Participant compensation

Parent participants will receive a $30.00 electronic gift card (ie, e-gift card) for completion of the study questionnaires, with an additional $20.00 e-gift card for participation in the qualitative interview (up to $50.00 total). Healthcare professional participants will receive a $50.00 e-gift card for participation in the qualitative interview.

#### Patient and public involvement

Patient partners (two NICU parents) acted as collaborators on the successful grant submission to the Canadian Institutes of Health Research. They enacted their participation on the research team by providing a letter of support endorsing the project’s expected benefits and outcomes. They were involved in the co-design of the study protocol. There was no public involvement in the project.

### Data collection

#### Quantitative data

All questionnaires will be administered through Research Electronic Data Capture (REDCap), a secure, web-based platform for building and managing research databases and surveys with built-in data validation and audit trails provided by the Research Institute of the McGill University Health Centre (RI-MUHC) for in-person or remote data collection.^52^ Participants will complete self-administered, computer-assisted questionnaires (see full list—table 1) via a tablet provided by the research team on-unit or remotely from the participant’s personal smartphone, electronic tablet or computer. The estimated time commitment is between 30 and 50 min for questionnaires. The selected measures include:

1. Demographics and Health Questionnaire, a set of items developed by the research team that captures participants’ medical history and sociodemographic profile.
2. Co-PARTNER Tool, a 31-item scale that measures parents’ active participation and collaboration with NICU HCPs.^53^
3. EMPATHIC-38-NICU-USA, a 38-item scale measuring parental satisfaction with NICU care.^54^
4. Maternal Postnatal Attachment Scale, a 19-item scale that measures the affective bond a mother develops with their newborn.^55^
5. Paternal Postnatal Attachment Scale, a 19-item scale that measures the affective bond a father/partner develops with their newborn.^56^
6. Multidimensional Scale of Perceived Social Support, a 12-item scale evaluating perceived support from family, friends and significant others. Widely used to assess the extent to which an individual feels socially supported.^57^
7. Parent Experience Questionnaire, a tool that evaluates parents’ early experiences and interactions with NICU services and evaluates their perceived quality of care and support.^58^
8. Parenting Sense of Competence Scale (PSOC), a 17-item instrument measuring parents’ confidence and satisfaction in caregiving tasks.^59^
9. Parenting Stress Scale, an 18-item questionnaire evaluating stress that is specifically associated with the parental role such as the demands of parenting, parental satisfaction and the perceived difficulties in interaction with the child.^60^
10. Perceived Stress Scale, a 10-item measure of general perceived stress. The scale consists of items that inquire about feelings and thoughts during the last month, with the aim of evaluating how unpredictable, uncontrollable and overloaded respondents find their lives.^61^
11. Parent Risk Evaluation and Engagement Model and Instrument, a 46-item qualitative and quantitative rating instrument used to assess parental fitness and child safety to help prioritise cases that are in need of services and determine the intensity of the services.^62^
12. Somatic Symptom Scale-8, an eight-item scale measuring the presence and severity of somatic symptoms related to stress.^63^
13. State-Trait Anxiety Inventory, a 20-item scale used to measure anxiety in adults, their current state of anxiety and trait anxiety.^64^

**Table 1:** Quantitative data collection

Questionnaire	Description	Maternal participant	Partner participant
Parental Well-Being			
Parenting Stress Scale (PSS)	Assesses both positive and negative aspects of parenting. Five-point Likert scale; higher scores=greater stress. Good internal consistency (α=0.83–0.86); test–retest reliability=0.81 over 6 weeks.^60^	X	X
Perceived Stress Scale (PSS-10)	Five-point Likert scale (0=never to 4=very often). Strong internal consistency (α=0.84–0.86); test–retest=0.85 (2 days), 0.55 (6 weeks). Correlates moderately with life events and depressive symptoms (r=0.18–0.36).^61^	X	X
Somatic Symptom Scale-8 (SSS-8)	Used in clinical and research settings to assess somatic symptom severity across four domains: gastrointestinal, pain, fatigue and cardiopulmonary. Strong internal reliability (α=0.81); correlates with depression (r=0.57), anxiety (r=0.55), and general health (r = −0.24).^63^	X	X
State-Trait Anxiety Inventory (STAI)	Used clinically to diagnose anxiety and distinguish it from depression; also measures caregiver distress. Rated on a four-point Likert scale (1=not at all to 4=very much). High internal consistency (α=0.86–0.95); test–retest reliability=0.65–0.75 (2 months). Supports good construct and concurrent validity.^64^	X	X
Parental Engagement and Collaboration with Healthcare Providers			
The EMPATHIC-38-NICU-USA Questionnaire	Assesses communication, professionalism and organisational aspects of the care unit. High internal reliability (α=0.90); domain-to-total correlations=0.38–0.91, indicating good validity.^54^	X	X
Parent Risk Evaluation and Engagement Model and Instrument (PREEMI)	Covers parental behaviour, environment and parent–child interactions across subscales (eg, attitudes, behavioural/environmental risks and child welfare engagement). High inter-rater reliability; incorporates multiple informants for cross-validation.^62^	X	X
Co-PARTNER Tool	Assesses parents’ roles in care, leadership and bonding with their infant. Internal consistency varies across domains (α=0.56–0.94). Strong convergent, discriminant, construct and structural validity.^53^	X	X
Parent Self-Efficacy			
Parent Experience Questionnaire (PEQ)	Explores early parent–clinician communication, intervention services, efficacy and barriers to supporting child development and parental experience. Uses five-point and seven-point Likert scales. Shows good feasibility, stability and association with parental mental health and child outcomes.^58^	X	X
Parenting Sense of Competence Scale (PSOC)	Measures two dimensions of parenting: satisfaction (emotional strain) and efficacy (perceived competence). Uses a six-point Likert scale. Good internal consistency (α=0.79).^59^	X	X
Maternal Postnatal Attachment Scale (MPAS)	Assesses maternal attachment quality, including warmth, absence of hostility and enjoyment in interaction. Uses a 2–5 point scale; higher scores=stronger attachment. Good internal consistency (α=0.70–0.78) and construct validity.^55^	X	
Paternal Postnatal Attachment Scale (PPAS)	Captures the father’s/partner’s feelings of comfort, affection and closeness to the infant. Adapted from MPAS to reflect the paternal experience. Uses a 2–5 point scale; higher scores=stronger attachment.^56^		X
Control Variables			
Demographics and Health Questionnaire	Evaluates demographic information such as cultural background, gender-identity, socio-economic status, educational attainment, relationship status, employment, postal code, etc.	X	X
Multidimensional Scale of Perceived Social Support (MSPSS)	Uses a seven-point Likert scale to assess perceived social support from family, friends and significant others. Strong internal consistency (α=0.81–0.98); factor structure confirmed. Validity supported by significant Multivariate Analysis of Variance (MANOVA) results.^57^	X	X

#### Qualitative data

The qualitative interviews will be conducted virtually or in-person by members of the research staff. The research staff will be taking part in a training session on how to conduct the interview beforehand. Maternal or partner participants will participate in one 60-minute qualitative interview session, and HCP participants will optionally participate in one 60-minute qualitative interview. The qualitative interviews will be conducted in person at the Montreal Children’s Hospital’s NICU or held via a videoconferencing platform (ie, Teams). Participants will be contacted with different time slot options at which they can schedule the qualitative interview session. Participants will then be emailed with confirmation of their chosen time slot (along with their link for the interview, if opted for remote). Instructions and reiterations of the purpose and nature of the interview will also be included in the email. Before beginning the session, participants will be provided with a brief overview of the interview, emphasising voluntary participation and the right to withdraw/stop at any point in time.

### Statistical and data analyses

#### Quantitative data analysis

Quantitative data will be analysed using descriptive and inferential statistics in RStudio, with a two-sided significance threshold set at p<0.05. Data will be assessed for normality, outliers and missingness. Sensitivity analyses will be conducted by excluding outliers, using multiple imputations for missing data, and conducting subgroup analyses (eg, by parent language or infant medical complexity).

Objective 1: Evaluate factors that promote parental engagement, self-efficacy, and collaboration with providers (Hypothesis 2)

Multiple regression analyses will be conducted to assess how variables such as emotional regulation, parental well-being and perceived provider support predict levels of parental self-efficacy and engagement.

Objective 2: Evaluate the role of healthcare provider collaboration in supporting parental self-efficacy and engagement (Hypothesis 4)

Pearson correlations and hierarchical regression models will be used to examine the relationship between parent-reported collaboration with providers and their reported self-efficacy and engagement in care.

#### Qualitative data analysis

Qualitative data will be coded using NVivo 12, using a grounded theory approach to identify key themes in parent and provider experiences.^65^

Objective 3: Identify the psychosocial and emotional needs of NICU parents (Hypothesis 1)

Thematic analysis of parent interviews will explore emotional responses, perceived stressors, coping strategies and unmet psychosocial needs during the NICU stay.

Objective 4: Identify barriers to parental engagement (Hypothesis 3)

Both parent and HCP interviews will be analysed to identify perceived structural, interpersonal and emotional barriers that may hinder parent involvement in infant care.

Triangulation will be used to integrate quantitative and qualitative findings, enhancing the depth and contextual understanding of how various factors influence parental engagement, self-efficacy and collaboration in NICU care. Qualitative findings will be used to interpret and elaborate on quantitative results, especially where unexpected patterns or subgroup differences emerge.

## Discussion and implication of findings

This study protocol highlights the critical role of parental self-efficacy in shaping meaningful engagement and collaboration with HCPs within the NICU context. Based on the existing body of literature, higher levels of self-efficacy are expected to be associated with increased parental engagement in infant care activities during the NICU stay and more effective communication with HCPs, suggesting a potential mechanism for improving both family experience and clinical outcomes.^66^ Grunberg *et al* demonstrated that parental self-efficacy significantly mediated the relationship between parent–staff interactions and both bonding and reduced psychological distress (eg, depression, anxiety) in NICU settings (b=–0.26 for bonding; b=–0.05 for depression).^67^ Similarly, Garfield *et al* found that targeted interventions designed to bolster self-efficacy led to sustained increases in parenting confidence—as measured by the PSOC scale—which were associated with greater engagement and improved communication with clinical teams (estimate=4.3 points higher; p=0.0042).^42^

Addressing parental psychosocial needs—such as stress, anxiety and uncertainty while in the NICU—may be essential for enhancing this self-efficacy.^68 69^ By identifying and addressing these needs at an early stage, NICUs can implement tailored interventions, such as peer support initiatives, caregiver coaching and structured communication strategies, to empower parents, promote their active engagement in care and ultimately enhance outcomes for both infants and their families.^15 17^ Importantly, improved parental engagement may not only support parent-infant bonding but also contribute to shorter hospital stays, thereby optimising health outcomes and healthcare resource utilisation.^15 24 68 70 71^

To address these findings, NICUs should move towards a more family-integrated model of care that recognises psychosocial support as a clinical priority. Embedding structured, parent-focused interventions into routine NICU practice may help foster developmentally supportive environments for both infants and families.^24 72 73^ Future research should explore scalable, evidence-based approaches to support parental confidence, particularly across diverse clinical and sociodemographic contexts, to ensure that all families receive adequate support during this critical transition.

## Ethics and dissemination

### Ethics and data privacy

This study protocol received approval from the Montreal University Health Centre’s (MUHC) Research Ethics Board (2025-9392) and adheres to the ethical standards of the Declaration of Helsinki (Article 8, 20–22, 2013) and the Tri-Council Policy Statement: Ethical Conduct for Research Involving Humans (TCPS 2).^74 75^ Parents exhibiting signs of distress during the study will be offered psychosocial support resources. HCPs that participate in the study will assist with parent recruitment; however, their involvement will remain confidential and not be disclosed to any NICU parents unless voluntarily shared.

#### Informed consent

Participants will complete an electronic informed consent form via REDCap and will replicate the physical copy with an option to contact the research team with questions or concerns. Participants may be contacted after their interview to verify the accuracy of their transcript, receive a summary of study findings or be invited to participate in future related research. Consent may be withdrawn at any time without consequence. Participants will confirm their consent by typing their name and checking a legal agreement box and will receive a PDF copy for their records. Any signed form will be stored securely on the REDCap server.

#### Data access and confidentiality

All data will be de-identified to maintain patient and provider confidentiality. Participant data will be stored on secure servers hosted by the RI-MUHC for up to 7 years following the completion of study recruitment. Afterwards, all data will then be destroyed. Any personal identifiers will be separated from the study data with linkage maintained only by the Principal Investigator (PI) and study coordinator. If data is transferred to other collaborating institutions, other lead researchers and co-investigators affiliated with the study. Secure servers and formal data transfer agreements will be used to maintain compliance with the confidentiality protocols.

Information collected from participants will remain strictly confidential within the limits provided by the law. Each participant will be assigned an identification number on recruitment so that their personal information and the information collected as a research subject will be stored in two separate databases. All study data will be collected using REDCap. Participants’ contact information and survey responses will be kept in the same project space but only accessible by the PI and study coordinator. REDCap will automatically flag and restrict access to identifiers and remove them from all exports unless explicitly permitted.

#### Audio/video recording and transcription

The qualitative interviews will be audio and/or video recorded when using a videoconferencing platform (Zoom or Teams) and when conducting the qualitative interviews in-person. In-person qualitative interviews will be video recorded using a digital camera. Data from the SD card of the camera will be transferred to the research team’s secured server housed and once uploaded to the server, it will immediately be deleted from the SD card. Each video recording will be saved using a file name that contains a random number and the date of the recording. A separate, password-protected Excel spreadsheet saved on the study’s secure server will link the video’s file name with the participant’s unique study ID. This Excel spreadsheet will only be accessible to the PI, the research coordinator and the research assistants responsible for carrying out the qualitative interviews. Additionally, only the PI, research coordinator and research assistants responsible for transcription of the videos will have access to the folder where the video recordings are saved. Trained research personnel will transcribe the interview via REDCap. The transcription on REDCap will be associated with the participant’s unique study ID and will not contain any identifiable information. Video recordings will be destroyed 7 years from the date of completion of recruitment for the study.

### Dissemination

Knowledge mobilisation will be carried out as end-of-grant dissemination activities and will include the preparation and writing of papers, as well as clinical rounds, conference presentations and media op-eds. The knowledge findings derived from this larger research project implementation have the potential to promote parental engagement in the NICU as a key predictor of family resilience and associated child more immediate and long-term well-being. Early life adversity has implications for longer term global functioning into adulthood, thus attesting to the importance and timeliness of this research.

## Supplementary Material

Reviewer comments

Author's manuscript
