# Supplementary material for: Optimising parental self-efficacy in the neonatal intensive care unit (NICU) and its implications on child care and parenting quality: a study protocol
Source: BMJ Open. 2026 May 25;16(5):e116985. doi: 10.1136/bmjopen-2026-116985 (PMC13223963; doi:10.1136/bmjopen-2026-116985)
Supplement: Author's manuscript [file bmjopen-2026-116985.draft_revisions.pdf]

BMJ Open is committed to open peer review. As part of this commitment we make the peer review history of every article we publish publicly available.

When an article is published we post the peer reviewers' comments and the authors' responses online. We also post the versions of the paper that were used during peer review. These are the versions that the peer review comments apply to.

The versions of the paper that follow are the versions that were submitted during the peer review process. They are not the versions of record or the final published versions. They should not be cited or distributed as the published version of this manuscript.

BMJ Open is an open access journal and the full, final, typeset and author-corrected version of record of the manuscript is available on our site with no access controls, subscription charges or pay-per-view fees (http://bmjopen.bmj.com).

If you have any questions on BMJ Open's open peer review process please email info.bmjopen@bmj.com

BMJ Open

Optimizing Parental Self-Efficacy in the Neonatal Intensive Care Unit (NICU): Implications on Child Care and Parenting Quality

Journal:	*BMJ Open*
Manuscript ID	bmjopen-2026-116985
Article Type:	Protocol
Date Submitted by the Author:	19-Jan-2026
Complete List of Authors:	Montreuil, Tina; McGill University, Department of Educational and Counselling Psychology; McGill University Health Centre, Research Institute; McGill University, Department of Psychiatry; McGill University, Department of Pediatrics<br>Gratton, Chloé; McGill University Department of Psychiatry, Psychiatry; Research Institute of the McGill University Health Centre, Child Health and Human Development<br>Mir-Shokraei, Kimia ; University of Waterloo, Department of Health Informatics<br>Biran, Valérie; Hopital Universitaire Robert Debre, Neonatal Intensive Care Unit; INSERM U1141,<br>Beltempo, Marc; McGill University, Pediatrics<br>Brossard-Racine, Marie; RI-MUHC<br>Lecorguillé, Marion ; McGill University, Department of Educational and Counselling Psychology; McGill University Health Centre, Research Institute
Keywords:	Neonatal intensive & critical care < INTENSIVE & CRITICAL CARE, Parents, Health, MENTAL HEALTH

SCHOLARONE™
MANUSCRIPTS

Optimizing Parental Self-Efficacy in the Neonatal Intensive Care Unit (NICU): Implications on Child Care and Parenting Quality

Tina C. Montreuil[1,2,3,4], Chloé Gratton[1], Kimia Mir-Shokraei[5], Valérie Biran[6], Marc Beltempo[7], Marie Brossard-Racine[2,8]*, Marion Lecorguillé[1,2]*
* equal contribution

1 Department of Educational and Counselling Psychology, Faculty of Education, McGill University, Montreal, QC, Canada.
2 Research Institute of the McGill University Health Centre, Montreal, QC, Canada.
3 Department of Psychiatry, Faculty of Medicine and Health Sciences, McGill University, Montreal, QC, Canada
4 Department of Pediatrics, Faculty of Medicine and Health Sciences, McGill University, Montreal, QC, Canada.
5 Department of Health Informatics, University of Waterloo, Waterloo, Ontario, Canada
6 Neonatal Care and Neonatal Resuscitation Service, Robert-Debré Hospital, Paris, France
7 Department of Pediatrics, Division of Neonatology, McGill University Health Centre, Montreal, QC, Canada
8 School of Physical & Occupational Therapy, McGill University, Montreal, QC, Canada

BMJ Open

Corresponding author: Tina C. Montreuil
Department of Educational and Counselling Psychology, McGill University, Montreal, QC, Canada.
Email: tina.montreuil@mcgill.ca

Funding: This work was supported by the Canadian Institute of Health Research (CIHR):

Planning and Dissemination Grant and the Montreal Children's Hospital Foundation.

Article Type: Protocol study

Word Count: 4,306

ABSTRACT

Introduction: Parenting an infant in the Neonatal Intensive Care Unit (NICU) can be highly stressful, undermine parental confidence, and minimize involvement in infant care. Enhancing parental self-efficacy is therefore crucial to promoting active engagement and supporting positive child outcomes. This study aims to identify the psychosocial needs (emotion regulation and stress management) of NICU parents and examine the factors that influence their engagement in infant care. It also seeks to determine whether higher levels of parental self-efficacy are associated with greater engagement and collaboration with healthcare providers (HCPs), and a shorter NICU stay.

Methods and Analysis: A mixed methods study will be conducted at the Montreal Children's Hospital's Level III NICU located in Quebec, Canada. Parents (i.e., mothers and, when available, their partners) will be recruited during their infant's hospitalization. Participants will complete a set of self-report questionnaires measuring parental engagement, self-efficacy, parent-infant attachment, mental health, perceived stress, and social support. Additionally, one parent per family will be invited to participate in a qualitative interview to discuss their experience in the NICU. Qualitative data will be analyzed thematically using NVivo 12 to identify key themes, and statistical analyses will be conducted using RStudio to determine which factors are most strongly associated with parental engagement and their interactions with the clinical team. Finally, healthcare providers will be invited to assist and participate in a qualitative interview regarding their perspectives on the NICU environment and the study's impact on the unit (i.e., reduction in healthcare provider burden due to increased parental engagement). Findings from this study will offer valuable insights to inform future clinical strategies aimed at enhancing parental engagement and self-efficacy in the NICU. These outcomes may contribute to improved neonatal outcomes, parental mental health, and the advancement of evidence-based, family-centered care policies.

Discussion and Implications of Findings: Our study protocol aims to underscore the critical role of self-efficacy and other key psychosocial factors, such as lower parental stress and higher emotion regulation, in promoting parental engagement and collaboration within the NICU. We anticipate that higher levels of self-efficacy will correlate with parental well-being, increased caregiving engagement (i.e., basic baby care, improved communication with NICU staff), and enhanced parent-infant bonding. These findings may support the development of structured interventions that empower parents and streamline provider-parent collaboration. In clinical practice, these insights can guide the integration of tailored parental support programs to optimize discharge preparation and reduce provider burden by promoting shared caregiving responsibilities. Future initiatives could build further on this model to guide institutional policies that aim to prioritize parental empowerment (i.e., greater self-efficacy and engagement) as a fundamental pillar of neonatal care.

Keywords: NICU, parental, engagement, self-efficacy, well-being, healthcare provider

STRENGTHS AND LIMITATIONS OF THE STUDY

- Mixed-methods design combining quantitative and qualitative approaches to provide comprehensive insights into the psychosocial needs, caregiving experiences, and perceived barriers faced by NICU parents.
- Use of validated instruments to assess parental engagement, self-efficacy, and mental health.
- Inclusion of both mothers and partners to capture dyadic experiences.
- Single-center study may limit generalizability beyond the pilot implementation institution.
- No post-discharge follow-up limits the understanding of long-term impacts on parents' and children's outcomes.

For peer review only - http://bmjopen.bmj.com/site/about/guidelines.xhtml

INTRODUCTION

Early life experiences play a pivotal role in shaping neurodevelopmental trajectories, particularly when adversity is faced during the critical periods of brain maturation[1]. Challenges occurring during sensitive windows of brain development—such as premature exposure to the extra-uterine environment—have been linked to long-term disruptions in cognitive, social-emotional, and neural functioning[2].

Admission of an infant to the Neonatal Intensive Care Unit (NICU) is inherently stressful and often elicits acute psychological distress in most parents, which may evolve into post-traumatic stress disorders, anxiety and depression[3]. This distress is intensified by NICU admission, typically prompted by acute or severe illness, where parents must also cope with the profound fear of losing their child. Parents tend to rely on certain coping styles to avoid becoming overwhelmed by perceived or actual threats to their child (i.e., impairments or death), compounded by their feelings of own competence as a parent[4]. Up to 35% of mothers and 24% of fathers report developing acute stress symptoms shortly after admission and, for many cases, persist well beyond discharge [5–7]. Pre-existing maternal mental health conditions, such as anxiety or depression during pregnancy, may further increase the likelihood of a NICU admission and exacerbate the risk of postpartum mental illness[8–11]. The NICU context, characterized by physical separation, complex medical technology, and dependence on healthcare providers' support, can be disruptive to the natural bonding and attachment processes. Consequently, many parents are left feeling unprepared and undermining their sense of competence[12–14].

Promoting Parental Well-Being in the NICU

For peer review only - http://bmjopen.bmj.com/site/about/guidelines.xhtml

Healthcare providers (HCPs) increasingly recognize the urgent need to address and support parental mental health in the NICU, while also fostering active parental engagement and attachment with the infant throughout hospitalization and beyond [13]. Parental engagement in the NICU refers to the active involvement of parents in their infant's direct caregiving activities (i.e., feeding, diapering, skin-to-skin, etc.), emotional bonding (i.e., eye contact and soothing), and collaborative decision-making with the NICU staff about treatment and discharge planning [15,16]. Engagement extends beyond physical presence during hospitalization; it reflects the degree to which parents participate in caregiving, communication, and collaboration with the healthcare team in the NICU. Higher engagement has been shown to bridge gaps across the multiple providers and services that infants often require and underpin successful transitions to home while reducing preventable readmissions [17,18]. However, emotional distress, the short length of NICU stays, and competing demands are limiting factors for meaningful engagement[13]. Understanding the emotional and psychological factors that promote, or limit engagement are therefore essential to better support families during their transition from hospital to home[19,20].

Within this framework, parental well-being can be promoted by targeting two key and interrelated processes: self-efficacy and emotion regulation. In the NICU context, parental sense of competence or self-efficacy refers to a parent's belief in their ability to successfully carry out caregiving tasks and effectively respond to their infant's needs, despite the stress and challenges of the intensive care environment [21,22]. It is rooted in Bandura's self-efficacy theory and is a key psychological resource for parents of preterm or medically fragile infants [23]. Strengthening parental self-efficacy early in the NICU has been shown to foster more meaningful engagement, reduce parental distress, and support healthier developmental outcomes[24]. In parallel, emotion regulation, defined as the ability to monitor, recognize and regulate one's emotional responses to

stressful events, is equally critical for parents in the NICU environment [25–27]. Unlike self-efficacy, emotion regulation relates to the parents' ability to manage distress and reframe negative thoughts while remaining responsive to their infant's cues among ongoing stressors[28]. This regulatory capacity allows for parents to stay present and aware during medical decision making, take part in nurturing interactions even when experiencing fear or grief, and avoid disengagement or withdrawal that can arise from overwhelming anxiety or traumatic stress[28,29]. Higher levels of emotion regulation not only preserves parental mental health but also strengthens the consistency and quality of parent-infant bonding[24,27].

Despite the recognized importance of engagement, parents continue to encounter accessibility and systemic challenges such as restrictive visiting hours, lack of on-site accommodation, challenges balancing work or caring for other children, and emotional distress; all of which might constrain their ability to fully participate in NICU caregiving[30–32]. Structured evidence-based early intervention programs for parental support remain inconsistently implemented, limiting many families' access to aid and resources to navigate their time in the NICU[33–38]. Moreover, most psychosocial interventions in the NICU remain disproportionately focused on mothers[3]. Fathers, although equally vulnerable to distress, are often underrepresented in studies and underserved in practice[39,40]. Emotional and psychological support must be actively integrated into NICU care to ensure that all parents are equipped to engage meaningfully in their infant's care, particularly those who remain underserved in both research and clinical practice[13,41,42]. Importantly, early interventions are consistent with growing evidence indicating that preventative approaches are both more effective and more cost-efficient than post-discharge care[43,44]. Given the multifaceted challenges that parents must face during NICU hospitalization, it is beneficial to examine how the

For peer review only - http://bmjopen.bmj.com/site/about/guidelines.xhtml

structure and delivery of care, including the role of healthcare providers, may contribute to mitigating these stressors.

Care Provider Role in Supporting Parental Well-Being

The NICU environment can be overwhelming and stressful for most parents, including those who do not typically exhibit emotional fragility or psychological distress under ordinary circumstances. Characterized by atypical lighting, medical emergency-related noises, and frequent medical procedure disruptions, the NICU environment presents daily challenges that may compromise parental well-being and, consequently, parent–infant bonding[3]. The physical immaturity of preterm infants, often following difficult, unplanned, or traumatic deliveries, may heighten parental distress and contribute to uncertainty about their baby's chances of survival. These stressors are compounded by factors beyond the hospital such as restricted visitation, caregiving demands at home, and emotional exhaustion. For new parents with pre-existing adverse childhood experiences or mental health conditions, recognizing these challenges is particularly critical as it can enable healthcare providers to implement timely, trauma-informed interventions that may mitigate the negative impacts of NICU-related stress and support parental well-being[45].

Healthcare providers play a pivotal role in alleviating or aggravating these challenges[46]. Empathic communication, hands-on training, and emotionally intelligent care have been identified as key factors that enhance parental confidence and engagement[47]. However, care quality often varies due to differences in healthcare provider training, workload, and emotional capacity[48]. Increasingly, COVID-19 has greatly contributed to heightened levels of burnout and compassion fatigue among providers[49], thus negatively affecting the quality of patient care in the post-COVID era. Relatedly, given the current demands placed on healthcare providers in the post-COVID context, fostering greater parental self-efficacy in NICU care may also help alleviate the burden

For peer review only - http://bmjopen.bmj.com/site/about/guidelines.xhtml

of the NICU nursing staff. As such, critically assessing the lived experiences of healthcare providers, beyond those of knowledge users and parents, is of utmost importance to ensure the delivery of quality care during the challenging transition to parenthood.

Despite known benefits of healthcare provider support on patient well-being, little is understood about the specific psychosocial and emotional needs of parents during NICU admission, and the factors that either promote or hinder their engagement [50,51]. Additionally, gaps remain in understanding how parental engagement and self-efficacy relate to measurable outcomes, such as length of NICU stay and discharge readiness.

Research Objectives

The overarching goal of this study protocol is to improve our understanding of how to optimize parental self-efficacy and engagement in the NICU, and to inform of interventions that could enhance childcare and parenting quality after NICU discharge. To do such, there are four specific aims:

1) Identify the psychosocial and emotional needs of parents in the NICU;
2) Evaluate factors that promote self-efficacy, parental engagement (i.e. communication and collaboration with providers in neonatal care), and improve parental well-being in the NICU
3) Identify barriers to parental engagement and;
4) Evaluate the role of healthcare provider collaboration in supporting parent self-efficacy and engagement with infant care.

Based on these aims and the literature, the hypotheses are the following:

For peer review only - http://bmjopen.bmj.com/site/about/guidelines.xhtml

It is hypothesized that parents presenting higher emotion regulation skills will demonstrate greater parental engagement in childcare during their stay in the NICU (Hypothesis 1). Secondly, we hypothesize that parents demonstrating greater engagement in childcare during their stay in the NICU will show increased participation and collaboration with healthcare providers in neonatal care (Hypothesis 2). Thirdly, it is hypothesized that families exhibiting greater parental engagement will have children with shorter NICU stays and an earlier discharge, when accounting for medical conditions, in comparison to families who display poorer parental engagement (Hypothesis 3). Finally, it is hypothesized that increased levels of collaboration and support from healthcare providers will be significantly associated with greater parental self-efficacy and engagement during the NICU stay (Hypothesis 4).

METHODS AND ANALYSIS

Study Design

This research is designed as an exploratory mixed-methods study combining quantitative online surveys and qualitative interviews. This approach aims to capture a comprehensive understanding of parental self-efficacy, engagement in NICU care, and the contextual factors shaping parent–provider interactions.

The study will take place in the Neonatal Intensive Care Unit (NICU) of the Montreal Children’s Hospital (Quebec, Canada). Canadian Level III NICU units provide regional intensive care[52]. NICU parent participants will include mothers of hospitalized infants and their partners (if applicable). NICU healthcare providers from the same institution will be recruited, including neonatologists, NICU nurses, social workers, psychologists, lactation consultants, and other providers directly involved in neonatal care.

Participant Recruitment

Inclusion and Exclusion Criteria

Eligible parents must be 18 years or older, have an infant currently admitted to the Montreal Children's Hospital's NICU, hold a valid RAMQ number and provide informed consent in either English or French. Parents will be excluded if they do not meet the above-mentioned inclusion criteria or if their child is actively receiving end-of-life care, to avoid any additional emotional burden. Healthcare providers must be 18 years or older, currently employed or affiliated with the Montreal Children's Hospital's NICU and can provide informed consent in either English or French. Providers who do not meet the inclusion criteria will be excluded.

Recruitment Procedures

Parents (N=50) will be recruited through two strategies: (1) in-unit advertising posters and handouts with a QR code that links to a pre-screening survey, and (2) in-person recruitment via the Montreal Children's Hospital's NICU Clinic Coordinator, NICU staff referrals and trained members of the study's research team. Eligible parents who have scanned the QR code will receive the electronic-informed consent form (i.e., e-consent) and a set of self-report questionnaires via email.

Healthcare providers (N=15) will be recruited through two strategies: (1) email outreach and (2) in-person recruitment. The research team will email the Montreal Children's Hospital's NICU staff with an overview of the study and a participation link. Interested providers can access the e-consent and enrollment form directly through this email.

Participant Compensation

Parent participants will receive a $30.00 electronic gift card (i.e., e-gift card) for completion of the study questionnaires, with an additional $20.00 e-gift card for participation in

For peer review only - http://bmjopen.bmj.com/site/about/guidelines.xhtml

the qualitative interview (up to $50.00 total). Healthcare professional participants will receive a $50.00 e-gift card for participation in the qualitative interview.

Patient and public involvement

Patient partners (two NICU parents) acted as collaborators on the successful grant submission to the Canadian Institutes of Health Research (CIHR). They enacted their participation on the research team by providing a letter of support endorsing the project's expected benefits and outcomes. They were involved in the co-design of the study protocol. There was no public involvement in the project.

Data Collection

Quantitative Data

All questionnaires will be administered through REDCap (Research Electronic Data Capture), a secure, web-based platform for building and managing research databases and surveys with built-in data validation and audit trails provided by the Research Institute of the McGill University Health Centre (RI-MUHC) for in-person or remote data collection[53]. Participants will complete self-administered, computer-assisted questionnaires (see full list – Table 1) via a tablet provided by the research team on-unit or remotely from the participant's personal smartphone, electronic tablet or computer. The estimated time commitment is between 30 to 50 minutes for questionnaires. The selected measures include:

1. Demographics and Health Questionnaire, a set of items developed by the research team that captures participants' medical history and sociodemographic profile.
2. Co-PARTNER Tool, a 31-item scale that measures parents' active participation and collaboration with NICU healthcare providers[54].

3. EMPATHIC-38-NICU-USA, a 38-item scale measuring parental satisfaction with NICU care[55].
4. Maternal Postnatal Attachment Scale (MPAS), a 19-item scale that measures the affective bond a mother develops with their newborn[56].
5. Paternal Postnatal Attachment Scale (PPAS), a 19-item scale that measures the affective bond a father/partner develops with their newborn[57].
6. Multidimensional Scale of Perceived Social Support (MSPSS), a 12-item scale evaluating perceived support from family, friends, and significant others. Widely used to assess the extent to which an individual feels socially supported[58].
7. Parent Experience Questionnaire (PEQ), a tool that evaluates parents' early experiences and interactions with NICU services and evaluates their perceived quality of care and support[59].
8. Parenting Sense of Competence Scale (PSOC), a 17-item instrument measuring parents' confidence and satisfaction in caregiving tasks[60].
9. Parenting Stress Scale (PSS), an 18-item questionnaire evaluating stress that is specifically associated with the parental role such as the demands of parenting, parental satisfaction, and the perceived difficulties in interaction with the child[61].
10. Perceived Stress Scale (PSS-10), a 10-item measure of general perceived stress. The scale consists of items that inquire about feelings and thoughts during the last month, with the aim of evaluating how unpredictable, uncontrollable, and overloaded respondents find their lives[62].
11. Parent Risk Evaluation and Engagement Model and Instrument (PREEMI), a 46-item qualitative and quantitative rating instrument used to assess parental fitness and child

For peer review only - http://bmjopen.bmj.com/site/about/guidelines.xhtml

safety to help prioritize cases that in need of services and determine the intensity of the services[63].

12. Somatic Symptom Scale-8 (SSS-8), an 8-item scale measuring the presence and severity of somatic symptoms related to stress[64].

13. State-Trait Anxiety Inventory (STAI), a 20-item scale used to measure anxiety in adults, their current state of anxiety and trait anxiety[65].

Table 1: Quantitative Data Collection

Questionnaire	Description	Maternal Participant	Partner Participant
**Parental Well-Being**			
Parenting Stress Scale (PSS)	Assesses both positive and negative aspects of parenting. 5-point Likert scale; higher scores = greater stress. Good internal consistency (α = 0.83–0.86); test-retest reliability = 0.81 over 6 weeks[61].	X	X
Perceived Stress Scale (PSS-10)	5-point Likert scale (0 = never to 4 = very often). Strong internal consistency (α = 0.84–0.86); test-retest = 0.85 (2 days), 0.55 (6 weeks). Correlates moderately with life events and depressive symptoms (r = 0.18–0.36)[62].	X	X
Somatic Symptom Scale-8 (SSS-8)	Used in clinical and research settings to assess somatic symptom severity across 4 domains: gastrointestinal, pain, fatigue, and cardiopulmonary. Strong internal reliability (α = 0.81); correlates with depression (r = 0.57), anxiety (r = 0.55), and general health (r = −0.24)[64].	X	X
State-Trait Anxiety Inventory (STAI)	Used clinically to diagnose anxiety and distinguish it from depression; also measures caregiver distress. Rated on a 4-point Likert scale (1 = Not at all to 4 = Very much). High internal consistency (α = 0.86–0.95); test-retest reliability = 0.65–0.75 (2 months). Supports good construct and concurrent validity[65].	X	X
**Parental Engagement and Collaboration with Healthcare Providers**			
The EMPATHIC-38-NICU-USA Questionnaire	Assesses communication, professionalism, and organizational aspects of the care unit. High internal reliability (α = 0.90); domain-to-total correlations = 0.38–0.91, indicating good validity[55].	X	X
Parent Risk Evaluation and Engagement Model	Covers parental behavior, environment, and parent–child interactions across subscales (e.g., attitudes, behavioral/environmental risks, and child welfare	X	X

and Instrument (PREEMI)	engagement). High inter-rater reliability; incorporates multiple informants for cross-validation[63].		
Co-PARTNER Tool	Assesses parents' roles in care, leadership, and bonding with their infant. Internal consistency varies across domains (α = 0.56–0.94). Strong convergent, discriminant, construct, and structural validity[54].	X	X
**Parent Self-Efficacy**			
Parent Experience Questionnaire (PEQ)	Explores early parent–clinician communication, intervention services, efficacy, and barriers to supporting child development and parental experience. Uses 5- and 7-point Likert scales. Shows good feasibility, stability, and association with parental mental health and child outcomes[59].	X	X
Parenting Sense of Competence Scale (PSOC)	Measures two dimensions of parenting: satisfaction (emotional strain) and efficacy (perceived competence). Uses a 6-point Likert scale. Good internal consistency (α = 0.79)[60].	X	X
Maternal Postnatal Attachment Scale (MPAS)	Assesses maternal attachment quality, including warmth, absence of hostility, and enjoyment in interaction. Uses a 2–5 point scale; higher scores = stronger attachment. Good internal consistency (α = 0.70–0.78) and construct validity[56].	X	
Paternal Postnatal Attachment Scale (PPAS)	Captures the father's/partner's feelings of comfort, affection, and closeness to the infant. Adapted from MPAS to reflect the paternal experience. Uses a 2–5 point scale; higher scores = stronger attachment[57].		X
**Control Variables**			
Demographics and Health Questionnaire	Evaluates demographic information such as cultural background, gender-identity, socio-economic status, educational attainment, relationship status, employment, postal code, etc.	X	X
Multidimensionnel Scale Perceived Social Support (MSPSS)	Uses a 7-point Likert scale to assess perceived social support from family, friends, and significant others. Strong internal consistency (α = 0.81–0.98); factor structure confirmed. Validity supported by significant MANOVA results[58].	X	X

Qualitative Data

The qualitative interviews will be conducted virtually or in-person by members of the research staff. The research staff will be taking part in a training session on how to conduct the interview

beforehand. Maternal or partner participants will participate in one 60-minute qualitative interview session, and healthcare provider participants will optionally participate in one 60-minute qualitative interview. The qualitative interviews will be conducted in person at the Montreal Children's Hospital's NICU or held via a videoconferencing platform (i.e. Teams). Participants will be contacted with different time slots options at which they can schedule the qualitative interview session. Participants will then be emailed with confirmation of their chosen time slot (along with their link for the interview, if opted for remote). Instructions and reiterations of the purpose and nature of the interview will also be included in the email. Before beginning the session, participants will be provided with a brief overview of the interview, emphasizing voluntary participation and the right to withdraw/stop at any point in time.

Statistical and Data Analyses

Quantitative Data Analysis

Quantitative data will be analyzed using descriptive and inferential statistics in RStudio, with a two-sided significance threshold set at $p < 0.05$. Data will be assessed for normality, outliers, and missingness. Sensitivity analyses will be conducted by excluding outliers, using multiple imputations for missing data, and conducting subgroup analyses (e.g., by parent language or infant medical complexity).

Objective 1: Evaluate factors that promote parental engagement, self-efficacy, and collaboration with providers (Hypothesis 2)

Multiple regression analyses will be conducted to assess how variables such as emotional regulation, parental well-being, and perceived provider support predict levels of parental self-efficacy and engagement.

For peer review only - http://bmjopen.bmj.com/site/about/guidelines.xhtml

Objective 2: Evaluate the role of healthcare provider collaboration in supporting parental self-efficacy and engagement (Hypothesis 4)

Pearson correlations and hierarchical regression models will be used to examine the relationship between parent-reported collaboration with providers and their reported self-efficacy and engagement in care.

Qualitative Data Analysis

Qualitative data will be coded using NVivo 12, using a grounded theory approach to identify key themes in parent and provider experiences[66].

Objective 3: Identify the psychosocial and emotional needs of NICU parents (Hypothesis 1)

Thematic analysis of parent interviews will explore emotional responses, perceived stressors, coping strategies, and unmet psychosocial needs during the NICU stay.

Objective 4: Identify barriers to parental engagement (Hypothesis 3)

Both parent and healthcare provider interviews will be analyzed to identify perceived structural, interpersonal, and emotional barriers that may hinder parent involvement in infant care.

Triangulation will be used to integrate quantitative and qualitative findings, enhancing the depth and contextual understanding of how various factors influence parental engagement, self-efficacy, and collaboration in NICU care. Qualitative findings will be used to interpret and elaborate on quantitative results, especially where unexpected patterns or subgroup differences emerge.

For peer review only - http://bmjopen.bmj.com/site/about/guidelines.xhtml

DISCUSSION AND IMPLICATION OF FINDINGS

This study protocol highlights the critical role of parental self-efficacy in shaping meaningful engagement and collaboration with healthcare providers within the NICU context. Based on the existing body of literature, higher levels of self-efficacy are expected to be associated with increased parental engagement in infant care activities during the NICU stay, and more effective communication with healthcare providers, suggesting a potential mechanism for improving both family experience and clinical outcomes[67]. Grunberg et al. demonstrated that parental self-efficacy significantly mediated the relationship between parent–staff interactions and both bonding and reduced psychological distress (e.g., depression, anxiety) in NICU settings (b = –0.26 for bonding; b = –0.05 for depression)[68]. Similarly, Garfield et al. found that targeted interventions designed to bolster self-efficacy led to sustained increases in parenting confidence—as measured by the Parenting Sense of Competence scale—which were associated with greater engagement and improved communication with clinical teams (estimate = 4.3 points higher; P = 0.0042)[43].

Addressing parental psychosocial needs—such as stress, anxiety, and uncertainty while in the NICU—may be essential for enhancing this self-efficacy[69,70]. By identifying and addressing these needs at an early stage, NICUs can implement tailored interventions, such as peer support initiatives, caregiver coaching, and structured communication strategies; to empower parents, promote their active engagement in care, and ultimately enhance outcomes for both infants and their families[15,17]. Importantly, improved parental engagement may not only support parent-infant bonding, but also contribute to shorter hospital stays, thereby optimizing health outcomes and health care resource utilization[15,24,69,71,72].

For peer review only - http://bmjopen.bmj.com/site/about/guidelines.xhtml

To address these findings, NICUs should move towards a more family-integrated model of care that recognizes psychosocial support as a clinical priority. Embedding structured, parent-focused interventions into routine NICU practice may help foster developmentally supportive environments for both infants and families[24,73,74]. Future research should explore scalable, evidence-based approaches to support parental confidence, particularly across diverse clinical and sociodemographic contexts, to ensure that all families receive adequate support during this critical transition.

For peer review only - http://bmjopen.bmj.com/site/about/guidelines.xhtml

ETHICS AND DISSEMINATION

Ethics and Data Privacy

This study protocol received approval from the Montreal University Health Centre's (MUHC) Research Ethics Board (2025-9392) and adheres to the ethical standards of the Declaration of Helsinki (Article 8, 20-22, 2013) and the Tri-Council Policy Statement: Ethical Conduct for Research Involving Humans (TCPS 2)[75,76]. Parents exhibiting signs of distress during the study will be offered psychosocial support resources. Healthcare providers that participate in the study will assist with parent recruitment; however, their involvement will remain confidential and not be disclosed to any NICU parents unless voluntarily shared.

Informed Consent

Participants will complete an electronic informed consent form via REDCap and will replicate the physical copy with an option to contact the research team with questions or concerns. Participants may be contacted after their interview to verify the accuracy of their transcript, receive a summary of study findings, or be invited to participate in future related research. Consent may be withdrawn at any time without consequence. Participants will confirm their consent by typing their name and checking a legal agreement box and will receive a PDF copy for their records. Any signed form will be stored securely on the REDCap server.

Data Access and Confidentiality

All data will be de-identified to maintain patient and provider confidentiality. Participant data will be stored on secure servers hosted by the RI-MUHC for up to seven years following the completion of study recruitment. Afterwards, all data will then be destroyed. Any personal identifiers will be separated from the study data with linkage maintained only by the Principal

For peer review only - http://bmjopen.bmj.com/site/about/guidelines.xhtml

Investigator (PI) and study coordinator. If data is transferred to other collaborating institutions, other lead researchers and co-investigators affiliated with the study. Secure servers and formal data transfer agreements will be used to maintain compliance with the confidentiality protocols.

Information collected from participants will remain strictly confidential within the limits provided by the law. Each participant will be assigned an identification number upon recruitment so that their personal information and the information collected as a research subject will be stored in two separate databases. All study data will be collected using REDCap. Participants' contact information and survey responses will be kept in the same project space but only accessible by the PI and study coordinator. REDCap will automatically flag and restrict access to identifiers and remove them from all exports unless explicitly permitted.

Audio/Video Recording and Transcription

The qualitative interviews will be audio and/or video recorded when using a videoconferencing platform (Zoom or Teams) and when conducting the qualitative interviews in-person. In-person qualitative interviews will be videorecorded using a digital camera. Data from the SD card of the camera will be transferred to the research team's secured server housed and once uploaded to the server, it will immediately be deleted from the SD card. Each video recording will be saved using a file name that contains a random number and the date of the recording. A separate, password-protected Excel spreadsheet saved on the study's secure server will link the video's file name with the participant's unique study ID. This Excel spreadsheet will only be accessible to the PI, the research coordinator and the research assistants responsible for carrying out the qualitative interviews. Additionally, only the PI, research coordinator and research assistants responsible for transcription of the videos will have access to the folder where

the video recordings are saved. Trained research personnel will transcribe the interview via REDCap. The transcription on REDCap will be associated with the participant's unique study ID and will not contain any identifiable information. Video recordings will be destroyed seven years from the date of completion of recruitment for the study.

Dissemination

Knowledge mobilization will be carried out as end-of-grant dissemination activities and will include the preparation and writing of papers, as well as clinical rounds, conference presentations and media op-eds. The knowledge findings derived from this larger research project implementation have the potential to promote parental engagement in the NICU as a key predictor of family resilience and associated child more immediate and long-term well-being. Early life adversity has implications for longer term global functioning into adulthood, thus attesting to the importance and timeliness of this research.

For peer review only - http://bmjopen.bmj.com/site/about/guidelines.xhtml

REFERENCES

1. Gee DG. Neurodevelopmental mechanisms linking early experiences and mental health: Translating science to promote well-being among youth. Am Psychol. 2022;77(9):1033–45.

2. Spittle A, Treyvaud K. The role of early developmental intervention to influence neurobehavioral outcomes of children born preterm. Semin Perinatol. 2016 Dec;40(8):542–8.

3. van Wyk L, Majiza AP, Ely CSE, Singer LT. Psychological distress in the neonatal intensive care unit: a meta-review. Pediatr Res. 2024 Nov;96(6):1510–8.

4. Shaw RJ, Bernard RS, Storfer-Isser A, Rhine W, Horwitz SM. Parental Coping in the Neonatal Intensive Care Unit. J Clin Psychol Med Settings. 2013 Jun;20(2):135–42.

5. Laccetta G, Di Chiara M, De Nardo MC, Terrin G. Symptoms of post-traumatic stress disorder in parents of preterm newborns: A systematic review of interventions and prevention strategies. Front Psychiatry [Internet]. 2023 Mar 8 [cited 2025 Jun 4];14. Available from: https://www.frontiersin.org/journals/psychiatry/articles/10.3389/fpsyt.2023.998995/full

6. Caporali C, Pisoni C, Gasparini L, Ballante E, Zecca M, Orcesi S, et al. A global perspective on parental stress in the neonatal intensive care unit: a meta-analytic study. J Perinatol. 2020 Dec;40(12):1739–52.

7. Feudtner C, Walter JK, Faerber JA, Hill DL, Carroll KW, Mollen CJ, et al. Good-parent beliefs of parents of seriously ill children. JAMA Pediatr. 2015 Jan;169(1):39–47.

8. Greene MM, Rossman B, Patra K, Kratovil AL, Janes JE, Meier PP. Depression, Anxiety, and Perinatal-Specific Posttraumatic Distress in Mothers of Very Low Birth Weight Infants in the Neonatal Intensive Care Unit. J Dev Behav Pediatr. 2015 Jun;36(5):362.

9. Latendresse G, Wong B, Dyer J, Wilson B, Baksh L, Hogue C. Duration of Maternal Stress and Depression: Predictors of Newborn Admission to Neonatal Intensive Care Unit and Postpartum Depression. Nurs Res. 2015 Oct;64(5):331.

10. Vasa R, Eldeirawi K, Kuriakose VG, G JN, Newsom C, Bates J. Postpartum Depression in Mothers of Infants in Neonatal Intensive Care Unit: Risk Factors and Management Strategies. Am J Perinatol. 2013 Aug 5;31:425–34.

11. Beck CT, Watson S, Gable RK. Traumatic Childbirth and Its Aftermath: Is There Anything Positive? J Perinat Educ. 2018 Jun 1;27(3):175–84.

12. Haward MF, Lantos J, Janvier A, for the POST Group. Helping Parents Cope in the NICU. Pediatrics. 2020 Jun 1;145(6):e20193567.

13. Lean RE, Rogers CE, Paul RA, Gerstein ED. NICU Hospitalization: Long-Term Implications on Parenting and Child Behaviors. Curr Treat Options Pediatr. 2018 Mar;4(1):49–69.

14. Murdoch MR, Franck LS. Gaining confidence and perspective: a phenomenological study of mothers' lived experiences caring for infants at home after neonatal unit discharge. J Adv Nurs. 2012 Sep;68(9):2008–20.

15. O'Brien K, Robson K, Bracht M, Cruz M, Lui K, Alvaro R, et al. Effectiveness of Family Integrated Care in neonatal intensive care units on infant and parent outcomes: a multicentre, multinational, cluster-randomised controlled trial. Lancet Child Adolesc Health. 2018 Apr;2(4):245–54.

16. Umberger E, Canvasser J, Hall SL. Enhancing NICU parent engagement and empowerment. Semin Pediatr Surg. 2018 Feb;27(1):19–24.

17. Novak JL, Vittner D. Parent engagement in the NICU. J Neonatal Nurs. 2021 Aug 1;27(4):257–62.

18. Boykova M, Kenner C. Transition from hospital to home for parents of preterm infants. J Perinat Neonatal Nurs. 2012;26(1):81–7; quiz 88–9.

19. Holditch-Davis D, Miles MS. Mothers' stories about their experiences in the neonatal intensive care unit. Neonatal Netw NN. 2000 Apr;19(3):13–21.

20. Association of Women's Health, Obstetric and Neonatal Nurses. Assessment and Care of the Late Preterm Infant. J Obstet Gynecol Neonatal Nurs JOGNN. 2024 Sep;53(5):e1–62.

21. Albanese AM, Russo GR, Geller PA. The role of parental self-efficacy in parent and child well-being: A systematic review of associated outcomes. Child Care Health Dev. 2019 May;45(3):333–63.

22. Barnes CR, Adamson-Macedo EN. Perceived Maternal Parenting Self-Efficacy (PMP S-E) tool: development and validation with mothers of hospitalized preterm neonates. J Adv Nurs. 2007 Dec;60(5):550–60.

23. Bandura A. Self-efficacy: Toward a unifying theory of behavioral change. Psychol Rev. 1977;84(2):191–215.

24. Pineda R, Bender J, Hall B, Shabosky L, Annecca A, Smith J. Parent Participation in the Neonatal Intensive Care Unit: Predictors and Relationships to Neurobehavior and Developmental Outcomes. Early Hum Dev. 2018 Feb;117:32–8.

25. Silva N de F, Linhares MBM, Gaspardo CM. Stress and self-regulation behaviors in preterm neonates hospitalized at open-bay and single-family room Neonatal Intensive Care Unit. Infant Behav Dev. 2024 Sep 1;76:101951.

For peer review only - http://bmjopen.bmj.com/site/about/guidelines.xhtml

26. Rutherford HJV, Wallace NS, Laurent HK, Mayes LC. Emotion Regulation in Parenthood. Dev Rev DR. 2015 Jun 1;36:1–14.

27. Dell'Aversana V, Tofani M, Valente D. Emotional Regulation Interventions on Developmental Course for Preterm Children: A Systematic Review of Randomized Control Trials. Children. 2023 Mar 22;10(3):603.

28. Hamzallari O, Rosinski L, Petrenko A, Bridgett DJ. Mothers' Emotion Regulation and Negative Affect in Infants: The Role of Self-Efficacy and Knowledge of Parenting Practices. Children. 2022 Dec 31;10(1):85.

29. Siva N, Nayak BS, Edward S Lewis L, Velayudhan B, Noronha JA. Intervention strategies to enhance early mother-infant interaction at NICU - A systematic review. J Neonatal Nurs. 2025 Jun 1;31(3):101671.

30. Almalki M, Gildea A, Boyle B. Parents' experiences of family-centred care in neonatal intensive care units: A qualitative thematic synthesis. J Neonatal Nurs. 2025 Jun 1;31(3):101655.

31. McCarty DB, Ferrari RM, Golden S, Zvara BJ, Wilson WD, Shanahan ME. Identifying Facilitators and Barriers to Neonatal Intensive Care Unit Visitation in Mothers of Low Socioeconomic Status: A Qualitative Investigation. Children. 2024 Nov 16;11(11):1390.

32. Neu M, Klawetter S, Greenfield JC, Roybal K, Scott JL, Hwang SS. Mothers' Experiences in the NICU Before Family-Centered Care and in NICUs Where It Is the Standard of Care. Adv Neonatal Care Off J Natl Assoc Neonatal Nurses. 2020 Feb;20(1):68–79.

33. Almalki M, Gildea A, Boyle B. Parents' experiences of family-centred care in neonatal intensive care units: A qualitative thematic synthesis. J Neonatal Nurs. 2025 Jun 1;31(3):101655.

34. Garrand S, Sherman N, Rentchier D, Jung AL. A Pa rent-to-Parent Program. Fam Community Health. 1978 Nov;1(3):103.

35. Dahan S, Bourque CJ, Reichherzer M, Prince J, Mantha G, Savaria M, et al. Community, Hope, and Resilience: Parental Perspectives on Peer Support in Neonatology. J Pediatr. 2022 Apr 1;243:85-90.e2.

36. Breastfeeding Peer Counselors as Direct Lactation Care Providers in the Neonatal Intensive Care Unit - Paula P. Meier, Janet L. Engstrom, Beverly Rossman, 2013 [Internet]. [cited 2025 Jul 1]. Available from: https://journals.sagepub.com/doi/abs/10.1177/0890334413482184

37. Mangurten HH, Slade C, Fitzsimons D. Parent-Parent Support in the Care of High-Risk Newborns. J Obstet Gynecol Neonatal Nurs. 1979;8(5):275–7.

38. Treyvaud K. Parent and family outcomes following very preterm or very low birth weight birth: A review. Semin Fetal Neonatal Med. 2014 Apr 1;19(2):131–5.

For peer review only - http://bmjopen.bmj.com/site/about/guidelines.xhtml

39. Toral-López I, Fernández-Alcántara M, González-Carrión P, Cruz-Quintana F, Rivas-Campos A, Pérez-Marfil N. Needs Perceived by Parents of Preterm Infants: Integrating Care Into the Early Discharge Process. J Pediatr Nurs Nurs Care Child Fam. 2016 Mar 1;31(2):e99–108.

40. Hearn G, Clarkson G, Day M. The Role of the NICU in Father Involvement, Beliefs, and Confidence: A Follow-up Qualitative Study. Adv Neonatal Care. 2020 Feb;20(1):80.

41. Madigan S, Atkinson L, Laurin K, Benoit D. Attachment and internalizing behavior in early childhood: A meta-analysis. Dev Psychol. 2013;49(4):672–89.

42. Ardila A, Rosselli M, Matute E, Inozemtseva O. Gender differences in cognitive development. Dev Psychol. 2011;47(4):984–90.

43. Garfield CF, Kerrigan E, Christie R, Jackson KL, Lee YS. A Mobile Health Intervention to Support Parenting Self-Efficacy in the Neonatal Intensive Care Unit from Admission to Home. J Pediatr. 2022 May 1;244:92–100.

44. Larocque C, Peterson WE, Squires JE, Mason-Ward M, Mayhew K, Harrison D. Family-centred care in the Neonatal Intensive Care Unit: A concept analysis and literature review. J Neonatal Nurs. 2021 Dec 1;27(6):402–11.

45. Malin KJ, Vittner D, Darilek U, McGlothen-Bell K, Crawford A, Koerner R, et al. Application of the Adverse Childhood Experiences Framework to the NICU. Adv Neonatal Care Off J Natl Assoc Neonatal Nurses. 2024 Feb 1;24(1):4–13.

46. Senechal E, Radeschi D, Kearney R, Shalish W, Sant'Anna G. Parents and Health Care Providers' Perspectives on Vital Signs Monitoring Technologies in the Neonatal Intensive Care Unit: An International Survey. Am J Perinatol. 2025 May 29;

47. Dall'Oglio I, Mascolo R, Portanova A, Ragni A, Amadio P, Fiori M, et al. Staff Perceptions of Family-Centered Care in Italian Neonatal Intensive Care Units: A Multicenter Cross-Sectional Study. Children. 2022 Sep;9(9):1401.

48. Lake ET, Hallowell SG, Kutney-Lee A, Hatfield LA, Del Guidice M, Boxer B, et al. Higher Quality of Care and Patient Safety Associated with Better NICU Work Environments. J Nurs Care Qual. 2016;31(1):24–32.

49. Lou NM, Montreuil T, Feldman LS, Fried GM, Lavoie-Tremblay M, Bhanji F, et al. Evaluations of Healthcare Providers' Perceived Support From Personal, Hospital, and System Resources: Implications for Well-Being and Management in Healthcare in Montreal, Quebec, During COVID-19. Eval Health Prof. 2021 Sep 1;44(3):319–22.

50. Ryynänen SP. Patient Process-Based Well-Being With the Support of a Close Person. J Patient Exp. 2023 Jan 1;10:23743735231155802.

51. Marzban S, Najafi M, Agolli A, Ashrafi E. Impact of Patient Engagement on Healthcare Quality: A Scoping Review. J Patient Exp. 2022 Jan 1;9:23743735221125439.

For peer review only - http://bmjopen.bmj.com/site/about/guidelines.xhtml

52. Rizzolo A, Shah PS, Bertelle V, Makary H, Ye XY, Abenhaim HA, et al. Association of timing of birth with mortality among preterm infants born in Canada. J Perinatol Off J Calif Perinat Assoc. 2021 Nov;41(11):2597–606.

53. Harris PA, Taylor R, Thielke R, Payne J, Gonzalez N, Conde JG. Research electronic data capture (REDCap)—A metadata-driven methodology and workflow process for providing translational research informatics support. J Biomed Inform. 2009 Apr 1;42(2):377–81.

54. Veenendaal NR van, Auxier JN, Schoor SRD van der, Franck LS, Stelwagen MA, Groof F de, et al. Development and psychometric evaluation of the CO-PARTNER tool for collaboration and parent participation in neonatal care. PLOS ONE. 2021 Jun 9;16(6):e0252074.

55. Lake ET, Smith JG, Staiger DO, Schoenauer KM, Rogowski JA. Measuring Parent Satisfaction With Care in Neonatal Intensive Care Units: The EMPATHIC-NICU-USA Questionnaire. Front Pediatr [Internet]. 2020 Oct 6 [cited 2025 Jun 4];8. Available from: https://www.frontiersin.org/journals/pediatrics/articles/10.3389/fped.2020.541573/full

56. Condon JT. Maternal Postnatal Attachment Scale [Measurement Instrument] [Internet]. 2015 [cited 2025 Jun 4]. Available from: https://doi.org/10.25957/5dc0f28d14338

57. Condon JT. Paternal Postnatal Attachment Scale [Measurement instrument] [Internet]. Flinders University. 2015 [cited 2025 Jun 4]. Available from: https://doi.org/10.25957/5dc0f8641433a

58. Zimet GD, Dahlem NW, Zimet SG, Farley GK. The Multidimensional Scale of Perceived Social Support. J Pers Assess. 1988;52(1):30–41.

59. Williams TS, McDonald KP, Roberts SD, Westmacott R, Ahola Kohut S, Dlamini N, et al. In their own words: developing the Parent Experiences Questionnaire following neonatal brain injury using participatory design. Brain Inj. 2018;32(11):1386–96.

60. Johnston C, and Mash EJ. A Measure of Parenting Satisfaction and Efficacy. J Clin Child Psychol. 1989 Jun 1;18(2):167–75.

61. Berry JO, Jones WH. The Parental Stress Scale: Initial Psychometric Evidence. J Soc Pers Relatsh. 1995 Aug 1;12(3):463–72.

62. Cohen S, Kamarck T, Mermelstein R. A Global Measure of Perceived Stress. J Health Soc Behav. 1983;24(4):385–96.

63. Samra HA, McGrath JM, Fischer S, Schumacher B, Dutcher J, Hansen J. The NICU Parent Risk Evaluation and Engagement Model and Instrument (PREEMI) for Neonates in Intensive Care Units. J Obstet Gynecol Neonatal Nurs. 2015 Jan 1;44(1):114–26.

64. Gierk B, Kohlmann S, Kroenke K, Spangenberg L, Zenger M, Brähler E, et al. The somatic symptom scale-8 (SSS-8): a brief measure of somatic symptom burden. JAMA Intern Med. 2014 Mar;174(3):399–407.

For peer review only - http://bmjopen.bmj.com/site/about/guidelines.xhtml

65. Spielberger CD. State-trait anxiety inventory: Bibliography, 2nd edn. Palo Alto. CA: Consulting Psychologists Press; 1989.

66. Lumivero. NVivo Qualitative Data Analysis Software [Internet]. 2017. Available from: www.lumivero.com

67. Guttmann KF, Raviv GN, Fortney CA, Ramirez M, Smith CB. Parent Perspectives on Communication Quality in the Neonatal Intensive Care Unit. Adv Neonatal Care Off J Natl Assoc Neonatal Nurses. 2024 Aug 1;24(4):382–8.

68. Grunberg VA, Presciutti A, Vranceanu AM, Lerou PH. Parental Self-Efficacy and Personal Time Help Explain Impact of Parent-Staff Interactions on Parental Distress and Bonding in the Neonatal Intensive Care Unit. J Pediatr. 2025 Jan;276:114300.

69. Melnyk BM, Feinstein NF, Alpert-Gillis L, Fairbanks E, Crean HF, Sinkin RA, et al. Reducing premature infants' length of stay and improving parents' mental health outcomes with the Creating Opportunities for Parent Empowerment (COPE) neonatal intensive care unit program: a randomized, controlled trial. Pediatrics. 2006 Nov;118(5):e1414-1427.

70. Turner M, Chur-Hansen A, Winefield H, Stanners M. The assessment of parental stress and support in the neonatal intensive care unit using the Parent Stress Scale - Neonatal Intensive Care Unit. Women Birth J Aust Coll Midwives. 2015 Sep;28(3):252–8.

71. Flacking R, Lehtonen L, Thomson G, Axelin A, Ahlqvist S, Moran VH, et al. Closeness and separation in neonatal intensive care. Acta Paediatr Oslo Nor 1992. 2012 Oct;101(10):1032–7.

72. Benzies KM, Magill-Evans JE, Hayden KA, Ballantyne M. Key components of early intervention programs for preterm infants and their parents: a systematic review and meta-analysis. BMC Pregnancy Childbirth. 2013;13 Suppl 1(Suppl 1):S10.

73. Johnson LC, McManus B, Blanchard Y, Nugent JK. The newborn behavioural observations system: A relationship-building intervention to support families in the neonatal intensive care unit. Acta Paediatr. 2025;114(4):752–9.

74. Franck LS, O'Brien K. The evolution of family-centered care: From supporting parent-delivered interventions to a model of family integrated care. Birth Defects Res. 2019 Sep;111(15):1044–59.

75. Government of Canada IAP on RE. Tri-Council Policy Statement: Ethical Conduct for Research Involving Humans – TCPS 2 (2022) [Internet]. 2023 [cited 2025 Jun 4]. Available from: https://ethics.gc.ca/eng/policy-politique_tcps2-eptc2_2022.html

76. WMA - The World Medical Association-WMA Declaration of Helsinki – Ethical Principles for Medical Research Involving Human Participants [Internet]. [cited 2025 Jun 4]. Available from: https://www.wma.net/policies-post/wma-declaration-of-helsinki/

For peer review only - http://bmjopen.bmj.com/site/about/guidelines.xhtml

AUTHOR CONTRIBUTIONS

Concept and design: T.C.M Funding acquisition: T.C.M.; M.B.R.. Drafting of the manuscript: T.C.M., K.M., C.G., M.L.. Critical revision of the manuscript for important intellectual content: T.C.M., K.M., C.G., M.B., M.L.. Manuscript review and editing: All authors meet the full authorship criteria.

FUNDING STATEMENT

This study protocol was funded via a Canadian Institutes of Health Research-CIHR (2023) Planning and Dissemination Grant (Grant #486178). Funds will support conducting interviews with parents and clinicians to collect data on lived experience, needs, and preference with respect to possible intervention, as well as the current barriers to developing a parental sense of personal self-efficacy, which are critical to the well-being of the developing child and new family.

CONFLICTS OF INTERESTS STATEMENT

The authors have no competing interests to declare.

ACKNOWLEDGEMENTS

The authors wish to thank the parents of the Montreal Children's Hospital NICU for their anticipated participation, openness and trust in sharing their experiences. They also wish to acknowledge the NICU staff and healthcare providers for their valuable support and collaboration in the facilitation of the study.

BMJ Open

Optimizing Parental Self-Efficacy in the Neonatal Intensive Care Unit (NICU) and its Implications on Child Care and Parenting Quality: A Study Protocol

Journal:	*BMJ Open*
Manuscript ID	bmjopen-2026-116985.R1
Article Type:	Protocol
Date Submitted by the Author:	22-Apr-2026
Complete List of Authors:	Montreuil, Tina; McGill University, Department of Educational and Counselling Psychology; McGill University Health Centre, Research Institute; McGill University, Department of Psychiatry; McGill University, Department of Pediatrics<br>Gratton, Chloé; McGill University Department of Psychiatry, Psychiatry; Research Institute of the McGill University Health Centre, Child Health and Human Development<br>Mir-Shokraei, Kimia ; University of Waterloo, Department of Health Informatics<br>Biran, Valérie; Hopital Universitaire Robert Debre, Neonatal Intensive Care Unit; Institut national de la santé et de la recherche médicale,<br>Beltempo, Marc; McGill University Faculty of Medicine, Pediatrics<br>Brossard-Racine, Marie; McGill University Faculty of Medicine<br>Lecorguillé, Marion ; McGill University, Department of Educational and Counselling Psychology; McGill University Health Centre, Research Institute
**Primary Subject Heading**:	Public health
Secondary Subject Heading:	Patient-centred medicine, Health services research
Keywords:	Neonatal intensive & critical care < INTENSIVE & CRITICAL CARE, Parents, Health, MENTAL HEALTH

SCHOLARONE MANUSCRIPTS

Optimizing Parental Self-Efficacy in the Neonatal Intensive Care Unit (NICU) and its Implications on Child Care and Parenting Quality: A Study Protocol

Tina C. Montreuil[1,2,3,4], Chloé Gratton[1], Kimia Mir-Shokraei[5], Valérie Biran[6], Marc Beltempo[7], Marie Brossard-Racine[2,8]*, Marion Lecorguillé[1,2]*
* equal contribution

1 Department of Educational and Counselling Psychology, Faculty of Education, McGill University, Montreal, QC, Canada.
2 Research Institute of the McGill University Health Centre, Montreal, QC, Canada.
3 Department of Psychiatry, Faculty of Medicine and Health Sciences, McGill University, Montreal, QC, Canada
4 Department of Pediatrics, Faculty of Medicine and Health Sciences, McGill University, Montreal, QC, Canada.
5 Department of Health Informatics, University of Waterloo, Waterloo, Ontario, Canada
6 Neonatal Care and Neonatal Resuscitation Service, Robert-Debré Hospital, Paris, France
7 Department of Pediatrics, Division of Neonatology, McGill University Health Centre, Montreal, QC, Canada
8 School of Physical & Occupational Therapy, McGill University, Montreal, QC, Canada

BMJ Open

Corresponding author: Tina C. Montreuil
Department of Educational and Counselling Psychology, McGill University, Montreal, QC, Canada.
Email: tina.montreuil@mcgill.ca

Funding: This work was supported by the Canadian Institute of Health Research (CIHR):

Planning and Dissemination Grant and the Montreal Children's Hospital Foundation.

Article Type: Protocol study

Word Count: 4,306

ABSTRACT

Introduction: Parenting an infant in the Neonatal Intensive Care Unit (NICU) can be highly stressful, undermine parental confidence, and minimize involvement in infant care. Enhancing parental self-efficacy is therefore crucial to promoting active engagement and supporting positive child outcomes. This study aims to identify the psychosocial needs (emotion regulation and stress management) of NICU parents and examine the factors that influence their engagement in infant care. It also seeks to determine whether higher levels of parental self-efficacy are associated with greater engagement and collaboration with healthcare providers (HCPs), and a shorter NICU stay.

Methods and Analysis: A mixed methods study will be conducted at the Montreal Children's Hospital's Level III NICU located in Quebec, Canada. Parents (i.e., mothers and, when available, their partners) will be recruited during their infant's hospitalization. Participants will complete a set of self-report questionnaires measuring parental engagement, self-efficacy, parent-infant attachment, mental health, perceived stress, and social support. Additionally, one parent per family will be invited to participate in a qualitative interview to discuss their experience in the NICU. Qualitative data will be analyzed thematically using NVivo 12 to identify key themes, and statistical analyses will be conducted using RStudio to determine which factors are most strongly associated with parental engagement and their interactions with the clinical team. Finally, healthcare providers will be invited to assist and participate in a qualitative interview regarding their perspectives on the NICU environment and the study's impact on the unit (i.e., reduction in healthcare provider burden due to increased parental engagement). Findings from this study will offer valuable insights to inform future clinical strategies aimed at enhancing parental engagement and self-efficacy in the NICU. These outcomes may contribute to improved neonatal outcomes, parental mental health, and the advancement of evidence-based, family-centered care policies.

Ethics and Dissemination: The protocol was approved by the Montreal University Health Centre's (MUHC) Research Ethics Board (2025-9392). Findings derived from this study have the potential to optimize parental engagement in the NICU to promote family resilience and child well-being.

Implications of Findings: Our study protocol aims to underscore the critical role of self-efficacy and other key psychosocial factors, such as lower parental stress and higher emotion regulation, in promoting parental engagement and collaboration within the NICU. We anticipate that higher levels of self-efficacy will correlate with parental well-being, increased caregiving engagement (i.e., basic baby care, improved communication with NICU staff), and enhanced parent-infant bonding. These findings may support the development of structured interventions that empower parents and streamline provider-parent collaboration. In clinical practice, these insights can guide the integration of tailored parental support programs to optimize discharge preparation and reduce provider burden by promoting shared caregiving responsibilities. Future initiatives could build further on this model to guide institutional policies that aim to prioritize parental empowerment (i.e., greater self-efficacy and engagement) as a fundamental pillar of neonatal care.

Keywords: NICU, parental, engagement, self-efficacy, well-being, healthcare provider

For peer review only - http://bmjopen.bmj.com/site/about/guidelines.xhtml

STRENGTHS AND LIMITATIONS OF THE STUDY

- Mixed-methods design combining quantitative and qualitative approaches to provide comprehensive insights into the psychosocial needs, caregiving experiences, and perceived barriers faced by NICU parents.
- Use of validated instruments to assess parental engagement, self-efficacy, and mental health.
- Inclusion of both mothers and partners to capture dyadic experiences.
- Single-center study may limit generalizability beyond the pilot implementation institution.
- No post-discharge follow-up limits the understanding of long-term impacts on parents' and children's outcomes.

For peer review only - http://bmjopen.bmj.com/site/about/guidelines.xhtml

INTRODUCTION

Early life experiences play a pivotal role in shaping neurodevelopmental trajectories, particularly when adversity is faced during the critical periods of brain maturation[1]. Challenges occurring during sensitive windows of brain development—such as premature exposure to the extra-uterine environment—have been linked to long-term disruptions in cognitive, social-emotional, and neural functioning[2].

Admission of an infant to the Neonatal Intensive Care Unit (NICU) is inherently stressful and often elicits acute psychological distress in most parents, which may evolve into post-traumatic stress disorders, anxiety and depression[3]. This distress is intensified by NICU admission, typically prompted by acute or severe illness, where parents must also cope with the profound fear of losing their child. Parents tend to rely on certain coping styles to avoid becoming overwhelmed by perceived or actual threats to their child (i.e., impairments or death), compounded by their feelings of own competence as a parent[4]. Up to 35% of mothers and 24% of fathers report developing acute stress symptoms shortly after admission and, for many cases, persist well beyond discharge [5–7]. Pre-existing maternal mental health conditions, such as anxiety or depression during pregnancy, may further increase the likelihood of a NICU admission and exacerbate the risk of postpartum mental illness[8–11]. The NICU context, characterized by physical separation, complex medical technology, and dependence on healthcare providers' support, can be disruptive to the natural bonding and attachment processes. Consequently, many parents are left feeling unprepared and undermining their sense of competence[12–14].

Promoting Parental Well-Being in the NICU

For peer review only - http://bmjopen.bmj.com/site/about/guidelines.xhtml

Healthcare providers (HCPs) increasingly recognize the urgent need to address and support parental mental health in the NICU, while also fostering active parental engagement and attachment with the infant throughout hospitalization and beyond [13]. Parental engagement in the NICU refers to the active involvement of parents in their infant's direct caregiving activities (i.e., feeding, diapering, skin-to-skin, etc.), emotional bonding (i.e., eye contact and soothing), and collaborative decision-making with the NICU staff about treatment and discharge planning [15,16]. Engagement extends beyond physical presence during hospitalization; it reflects the degree to which parents participate in caregiving, communication, and collaboration with the healthcare team in the NICU. Higher engagement has been shown to bridge gaps across the multiple providers and services that infants often require and underpin successful transitions to home while reducing preventable readmissions [17,18]. However, emotional distress, the short length of NICU stays, and competing demands are limiting factors for meaningful engagement[13]. Understanding the emotional and psychological factors that promote, or limit engagement are therefore essential to better support families during their transition from hospital to home[19,20].

Within this framework, parental well-being can be promoted by targeting two key and interrelated processes: self-efficacy and emotion regulation. In the NICU context, parental sense of competence or self-efficacy refers to a parent's belief in their ability to successfully carry out caregiving tasks and effectively respond to their infant's needs, despite the stress and challenges of the intensive care environment [21,22]. It is rooted in Bandura's self-efficacy theory and is a key psychological resource for parents of preterm or medically fragile infants [23]. Strengthening parental self-efficacy early in the NICU has been shown to foster more meaningful engagement, reduce parental distress, and support healthier developmental outcomes[24]. In parallel, emotion regulation, defined as the ability to monitor, recognize and regulate one's emotional responses to

For peer review only - http://bmjopen.bmj.com/site/about/guidelines.xhtml

stressful events, is equally critical for parents in the NICU environment [25–27]. Unlike self-efficacy, emotion regulation relates to the parents' ability to manage distress and reframe negative thoughts while remaining responsive to their infant's cues among ongoing stressors[28]. This regulatory capacity allows for parents to stay present and aware during medical decision making, take part in nurturing interactions even when experiencing fear or grief, and avoid disengagement or withdrawal that can arise from overwhelming anxiety or traumatic stress[28,29]. Higher levels of emotion regulation not only preserves parental mental health but also strengthens the consistency and quality of parent-infant bonding[24,27].

Despite the recognized importance of engagement, parents continue to encounter accessibility and systemic challenges such as restrictive visiting hours, lack of on-site accommodation, challenges balancing work or caring for other children, and emotional distress; all of which might constrain their ability to fully participate in NICU caregiving[30–32]. Structured evidence-based early intervention programs for parental support remain inconsistently implemented, limiting many families' access to aid and resources to navigate their time in the NICU[33–38]. Moreover, most psychosocial interventions in the NICU remain disproportionately focused on mothers[3]. Fathers, although equally vulnerable to distress, are often underrepresented in studies and underserved in practice[39,40]. Emotional and psychological support must be actively integrated into NICU care to ensure that all parents are equipped to engage meaningfully in their infant's care, particularly those who remain underserved in both research and clinical practice[13,41,42]. Importantly, early interventions are consistent with growing evidence indicating that preventative approaches are both more effective and more cost-efficient than post-discharge care[43,44]. Given the multifaceted challenges that parents must face during NICU hospitalization, it is beneficial to examine how the

For peer review only - http://bmjopen.bmj.com/site/about/guidelines.xhtml

structure and delivery of care, including the role of healthcare providers, may contribute to mitigating these stressors.

Care Provider Role in Supporting Parental Well-Being

The NICU environment can be overwhelming and stressful for most parents, including those who do not typically exhibit emotional fragility or psychological distress under ordinary circumstances. Characterized by atypical lighting, medical emergency-related noises, and frequent medical procedure disruptions, the NICU environment presents daily challenges that may compromise parental well-being and, consequently, parent–infant bonding[3]. The physical immaturity of preterm infants, often following difficult, unplanned, or traumatic deliveries, may heighten parental distress and contribute to uncertainty about their baby's chances of survival. These stressors are compounded by factors beyond the hospital such as restricted visitation, caregiving demands at home, and emotional exhaustion. For new parents with pre-existing adverse childhood experiences or mental health conditions, recognizing these challenges is particularly critical as it can enable healthcare providers to implement timely, trauma-informed interventions that may mitigate the negative impacts of NICU-related stress and support parental well-being[45].

Healthcare providers play a pivotal role in alleviating or aggravating these challenges[46]. Empathic communication, hands-on training, and emotionally intelligent care have been identified as key factors that enhance parental confidence and engagement[47]. However, care quality often varies due to differences in healthcare provider training, workload, and emotional capacity[48]. Increasingly, COVID-19 has greatly contributed to heightened levels of burnout and compassion fatigue among providers[49], thus negatively affecting the quality of patient care in the post-COVID era. Relatedly, given the current demands placed on healthcare providers in the post-COVID context, fostering greater parental self-efficacy in NICU care may also help alleviate the burden

For peer review only - http://bmjopen.bmj.com/site/about/guidelines.xhtml

of the NICU nursing staff. As such, critically assessing the lived experiences of healthcare providers, beyond those of knowledge users and parents, is of utmost importance to ensure the delivery of quality care during the challenging transition to parenthood.

Despite known benefits of healthcare provider support on patient well-being, little is understood about the specific psychosocial and emotional needs of parents during NICU admission, and the factors that either promote or hinder their engagement [50,51]. Additionally, gaps remain in understanding how parental engagement and self-efficacy relate to measurable outcomes, such as length of NICU stay and discharge readiness.

Research Objectives

The overarching goal of this study protocol is to improve our understanding of how to optimize parental self-efficacy and engagement in the NICU, and to inform interventions that could enhance childcare and parenting quality after NICU discharge. To do such, there are four specific aims:

1) Identify the psychosocial and emotional needs of parents in the NICU;
2) Evaluate factors that promote self-efficacy, parental engagement (i.e. communication and collaboration with providers in neonatal care), and improve parental well-being in the NICU
3) Identify barriers to parental engagement and;
4) Evaluate the role of healthcare provider collaboration in supporting parent self-efficacy and engagement with infant care.

Based on these aims and the literature, the hypotheses are the following:

For peer review only - http://bmjopen.bmj.com/site/about/guidelines.xhtml

It is hypothesized that parents presenting higher emotion regulation skills will demonstrate greater parental engagement in childcare during their stay in the NICU (Hypothesis 1). Secondly, we hypothesize that parents demonstrating greater engagement in childcare during their stay in the NICU will show increased participation and collaboration with healthcare providers in neonatal care (Hypothesis 2). Thirdly, it is hypothesized that families exhibiting greater parental engagement will have children with shorter NICU stays and an earlier discharge, when accounting for medical conditions, in comparison to families who display poorer parental engagement (Hypothesis 3). Finally, it is hypothesized that increased levels of collaboration and support from healthcare providers will be significantly associated with greater parental self-efficacy and engagement during the NICU stay (Hypothesis 4).

METHODS AND ANALYSIS

Study Design

This research is designed as an exploratory mixed-methods study combining quantitative online surveys and qualitative interviews. This approach aims to capture a comprehensive understanding of parental self-efficacy, engagement in NICU care, and the contextual factors shaping parent–provider interactions.

The study will take place in the Neonatal Intensive Care Unit (NICU) of the Montreal Children's Hospital (Quebec, Canada). Canadian Level III NICU units provide regional intensive care[52]. NICU parent participants will include mothers of hospitalized infants and their partners (if applicable). NICU healthcare providers from the same institution will be recruited, including neonatologists, NICU nurses, social workers, psychologists, lactation consultants, and other providers directly involved in neonatal care.

For peer review only - http://bmjopen.bmj.com/site/about/guidelines.xhtml

Participant Recruitment

Inclusion and Exclusion Criteria

Eligible parents must be 18 years or older, have an infant currently admitted to the Montreal Children's Hospital's NICU, hold a valid Régie de l'assurance maladie du Québec (RAMQ) number and provide informed consent in either English or French. Parents will be excluded if they do not meet the above-mentioned inclusion criteria or if their child is actively receiving end-of-life care, to avoid any additional emotional burden. Healthcare providers must be 18 years or older, currently employed or affiliated with the Montreal Children's Hospital's NICU and can provide informed consent in either English or French. Providers who do not meet the inclusion criteria will be excluded.

Recruitment Procedures

Parents (N=50) will be recruited through two strategies: (1) in-unit advertising posters and handouts with a QR code that links to a pre-screening survey, and (2) in-person recruitment via the Montreal Children's Hospital's NICU Clinic Coordinator, NICU staff referrals and trained members of the study's research team. Eligible parents who have scanned the QR code will receive the electronic-informed consent form (i.e., e-consent) and a set of self-report questionnaires via email.

Healthcare providers (N=15) will be recruited through two strategies: (1) email outreach and (2) in-person recruitment. The research team will email the Montreal Children's Hospital's NICU staff with an overview of the study and a participation link. Interested providers can access the e-consent and enrollment form directly through this email.

Participant Compensation

Parent participants will receive a $30.00 electronic gift card (i.e., e-gift card) for completion the study questionnaires, with an additional $20.00 e-gift card for participation in the qualitative interview (up to $50.00 total). Healthcare professional participants will receive a $50.00 e-gift card for participation in the qualitative interview.

Patient and public involvement

Patient partners (two NICU parents) acted as collaborators on the successful grant submission to the Canadian Institutes of Health Research (CIHR). They enacted their participation on the research team by providing a letter of support endorsing the project's expected benefits and outcomes. They were involved in the co-design of the study protocol. There was no public involvement in the project.

Data Collection

Quantitative Data

All questionnaires will be administered through REDCap (Research Electronic Data Capture), a secure, web-based platform for building and managing research databases and surveys with built-in data validation and audit trails provided by the Research Institute of the McGill University Health Centre (RI-MUHC) for in-person or remote data collection[53]. Participants will complete self-administered, computer-assisted questionnaires (see full list – Table 1) via a tablet provided by the research team on-unit or remotely from the participant's personal smartphone, electronic tablet or computer. The estimated time commitment is between 30 to 50 minutes for questionnaires. The selected measures include:

1. Demographics and Health Questionnaire, a set of items developed by the research team that captures participants' medical history and sociodemographic profile.

2. Co-PARTNER Tool, a 31-item scale that measures parents' active participation and collaboration with NICU healthcare providers[54].
3. EMPATHIC-38-NICU-USA, a 38-item scale measuring parental satisfaction with NICU care[55].
4. Maternal Postnatal Attachment Scale (MPAS), a 19-item scale that measures the affective bond a mother develops with their newborn[56].
5. Paternal Postnatal Attachment Scale (PPAS), a 19-item scale that measures the affective bond a father/partner develops with their newborn[57].
6. Multidimensional Scale of Perceived Social Support (MSPSS), a 12-item scale evaluating perceived support from family, friends, and significant others. Widely used to assess the extent to which an individual feels socially supported[58].
7. Parent Experience Questionnaire (PEQ), a tool that evaluates parents' early experiences and interactions with NICU services and evaluates their perceived quality of care and support[59].
8. Parenting Sense of Competence Scale (PSOC), a 17-item instrument measuring parents' confidence and satisfaction in caregiving tasks[60].
9. Parenting Stress Scale (PSS), an 18-item questionnaire evaluating stress that is specifically associated with the parental role such as the demands of parenting, parental satisfaction, and the perceived difficulties in interaction with the child[61].
10. Perceived Stress Scale (PSS-10), a 10-item measure of general perceived stress. The scale consists of items that inquire about feelings and thoughts during the last month, with the aim of evaluating how unpredictable, uncontrollable, and overloaded respondents find their lives[62].

For peer review only - http://bmjopen.bmj.com/site/about/guidelines.xhtml

11. Parent Risk Evaluation and Engagement Model and Instrument (PREEMI), a 46-item qualitative and quantitative rating instrument used to assess parental fitness and child safety to help prioritize cases that in need of services and determine the intensity of the services[63].
12. Somatic Symptom Scale-8 (SSS-8), an 8-item scale measuring the presence and severity of somatic symptoms related to stress[64].
13. State-Trait Anxiety Inventory (STAI), a 20-item scale used to measure anxiety in adults, their current state of anxiety and trait anxiety[65].

Table 1: Quantitative Data Collection

Questionnaire	Description	Maternal Participant	Partner Participant
**Parental Well-Being**			
Parenting Stress Scale (PSS)	Assesses both positive and negative aspects of parenting. 5-point Likert scale; higher scores = greater stress. Good internal consistency (α = 0.83–0.86); test-retest reliability = 0.81 over 6 weeks[61].	X	X
Perceived Stress Scale (PSS-10)	5-point Likert scale (0 = never to 4 = very often). Strong internal consistency (α = 0.84–0.86); test-retest = 0.85 (2 days), 0.55 (6 weeks). Correlates moderately with life events and depressive symptoms (r = 0.18–0.36)[62].	X	X
Somatic Symptom Scale-8 (SSS-8)	Used in clinical and research settings to assess somatic symptom severity across 4 domains: gastrointestinal, pain, fatigue, and cardiopulmonary. Strong internal reliability (α = 0.81); correlates with depression (r = 0.57), anxiety (r = 0.55), and general health (r = −0.24)[64].	X	X
State-Trait Anxiety Inventory (STAI)	Used clinically to diagnose anxiety and distinguish it from depression; also measures caregiver distress. Rated on a 4-point Likert scale (1 = Not at all to 4 = Very much). High internal consistency (α = 0.86–0.95); test-retest reliability = 0.65–0.75 (2 months). Supports good construct and concurrent validity[65].	X	X
**Parental Engagement and Collaboration with Healthcare Providers**			

For peer review only - http://bmjopen.bmj.com/site/about/guidelines.xhtml

The EMPATHIC-38-NICU-USA Questionnaire	Assesses communication, professionalism, and organizational aspects of the care unit. High internal reliability (α = 0.90); domain-to-total correlations = 0.38–0.91, indicating good validity[55].	X	X
Parent Risk Evaluation and Engagement Model and Instrument (PREEMI)	Covers parental behavior, environment, and parent–child interactions across subscales (e.g., attitudes, behavioral/environmental risks, and child welfare engagement). High inter-rater reliability; incorporates multiple informants for cross-validation[63].	X	X
Co-PARTNER Tool	Assesses parents' roles in care, leadership, and bonding with their infant. Internal consistency varies across domains (α = 0.56–0.94). Strong convergent, discriminant, construct, and structural validity[54].	X	X
**Parent Self-Efficacy**			
Parent Experience Questionnaire (PEQ)	Explores early parent–clinician communication, intervention services, efficacy, and barriers to supporting child development and parental experience. Uses 5- and 7-point Likert scales. Shows good feasibility, stability, and association with parental mental health and child outcomes[59].	X	X
Parenting Sense of Competence Scale (PSOC)	Measures two dimensions of parenting: satisfaction (emotional strain) and efficacy (perceived competence). Uses a 6-point Likert scale. Good internal consistency (α = 0.79)[60].	X	X
Maternal Postnatal Attachment Scale (MPAS)	Assesses maternal attachment quality, including warmth, absence of hostility, and enjoyment in interaction. Uses a 2–5 point scale; higher scores = stronger attachment. Good internal consistency (α = 0.70–0.78) and construct validity[56].	X	
Paternal Postnatal Attachment Scale (PPAS)	Captures the father's/partner's feelings of comfort, affection, and closeness to the infant. Adapted from MPAS to reflect the paternal experience. Uses a 2–5 point scale; higher scores = stronger attachment[57].		X
**Control Variables**			
Demographics and Health Questionnaire	Evaluates demographic information such as cultural background, gender-identity, socio-economic status, educational attainment, relationship status, employment, postal code, etc.	X	X
Multidimensionnel Scale Perceived Social Support (MSPSS)	Uses a 7-point Likert scale to assess perceived social support from family, friends, and significant others. Strong internal consistency (α = 0.81–0.98); factor structure confirmed. Validity supported by significant MANOVA results[58].	X	X

Qualitative Data

For peer review only - http://bmjopen.bmj.com/site/about/guidelines.xhtml

The qualitative interviews will be conducted virtually or in-person by members of the research staff. The research staff will be taking part in a training session on how to conduct the interview beforehand. Maternal or partner participants will participate in one 60-minute qualitative interview session, and healthcare provider participants will optionally participate in one 60-minute qualitative interview. The qualitative interviews will be conducted in person at the Montreal Children's Hospital's NICU or held via a videoconferencing platform (i.e. Teams). Participants will be contacted with different time slots options at which they can schedule the qualitative interview session. Participants will then be emailed with confirmation of their chosen time slot (along with their link for the interview, if opted for remote). Instructions and reiterations of the purpose and nature of the interview will also be included in the email. Before beginning the session, participants will be provided with a brief overview of the interview, emphasizing voluntary participation and the right to withdraw/stop at any point in time.

Statistical and Data Analyses

Quantitative Data Analysis

Quantitative data will be analyzed using descriptive and inferential statistics in RStudio, with a two-sided significance threshold set at $p < 0.05$. Data will be assessed for normality, outliers, and missingness. Sensitivity analyses will be conducted by excluding outliers, using multiple imputations for missing data, and conducting subgroup analyses (e.g., by parent language or infant medical complexity).

Objective 1: Evaluate factors that promote parental engagement, self-efficacy, and collaboration with providers (Hypothesis 2)

For peer review only - http://bmjopen.bmj.com/site/about/guidelines.xhtml

Multiple regression analyses will be conducted to assess how variables such as emotional regulation, parental well-being, and perceived provider support predict levels of parental self-efficacy and engagement.

Objective 2: Evaluate the role of healthcare provider collaboration in supporting parental self-efficacy and engagement (Hypothesis 4)

Pearson correlations and hierarchical regression models will be used to examine the relationship between parent-reported collaboration with providers and their reported self-efficacy and engagement in care.

Qualitative Data Analysis

Qualitative data will be coded using NVivo 12, using a grounded theory approach to identify key themes in parent and provider experiences[66].

Objective 3: Identify the psychosocial and emotional needs of NICU parents (Hypothesis 1)

Thematic analysis of parent interviews will explore emotional responses, perceived stressors, coping strategies, and unmet psychosocial needs during the NICU stay.

Objective 4: Identify barriers to parental engagement (Hypothesis 3)

Both parent and healthcare provider interviews will be analyzed to identify perceived structural, interpersonal, and emotional barriers that may hinder parent involvement in infant care.

Triangulation will be used to integrate quantitative and qualitative findings, enhancing the depth and contextual understanding of how various factors influence parental engagement, self-efficacy, and collaboration in NICU care. Qualitative findings will be used to interpret and

For peer review only - http://bmjopen.bmj.com/site/about/guidelines.xhtml

elaborate on quantitative results, especially where unexpected patterns or subgroup differences emerge.

DISCUSSION AND IMPLICATION OF FINDINGS

This study protocol highlights the critical role of parental self-efficacy in shaping meaningful engagement and collaboration with healthcare providers within the NICU context. Based on the existing body of literature, higher levels of self-efficacy are expected to be associated with increased parental engagement in infant care activities during the NICU stay, and more effective communication with healthcare providers, suggesting a potential mechanism for improving both family experience and clinical outcomes[67]. Grunberg et al. demonstrated that parental self-efficacy significantly mediated the relationship between parent–staff interactions and both bonding and reduced psychological distress (e.g., depression, anxiety) in NICU settings ($b = –0.26$ for bonding; $b = –0.05$ for depression)[68]. Similarly, Garfield et al. found that targeted interventions designed to bolster self-efficacy led to sustained increases in parenting confidence—as measured by the Parenting Sense of Competence scale—which were associated with greater engagement and improved communication with clinical teams (estimate = 4.3 points higher; $P = 0.0042$)[43].

Addressing parental psychosocial needs—such as stress, anxiety, and uncertainty while in the NICU—may be essential for enhancing this self-efficacy[69,70]. By identifying and addressing these needs at an early stage, NICUs can implement tailored interventions, such as peer support initiatives, caregiver coaching, and structured communication strategies; to empower parents, promote their active engagement in care, and ultimately enhance outcomes for both infants and their families[15,17]. Importantly, improved parental engagement may not only support parent-infant

For peer review only - http://bmjopen.bmj.com/site/about/guidelines.xhtml

bonding, but also contribute to shorter hospital stays, thereby optimizing health outcomes and health care resource utilization[15,24,69,71,72].

To address these findings, NICUs should move towards a more family-integrated model of care that recognizes psychosocial support as a clinical priority. Embedding structured, parent-focused interventions into routine NICU practice may help foster developmentally supportive environments for both infants and families[24,73,74]. Future research should explore scalable, evidence-based approaches to support parental confidence, particularly across diverse clinical and sociodemographic contexts, to ensure that all families receive adequate support during this critical transition.

For peer review only - http://bmjopen.bmj.com/site/about/guidelines.xhtml

ETHICS AND DISSEMINATION

Ethics and Data Privacy

This study protocol received approval from the Montreal University Health Centre's (MUHC) Research Ethics Board (2025-9392) and adheres to the ethical standards of the Declaration of Helsinki (Article 8, 20-22, 2013) and the Tri-Council Policy Statement: Ethical Conduct for Research Involving Humans (TCPS 2)[75,76]. Parents exhibiting signs of distress during the study will be offered psychosocial support resources. Healthcare providers that participate in the study will assist with parent recruitment; however, their involvement will remain confidential and not be disclosed to any NICU parents unless voluntarily shared.

Informed Consent

Participants will complete an electronic informed consent form via REDCap and will replicate the physical copy with an option to contact the research team with questions or concerns. Participants may be contacted after their interview to verify the accuracy of their transcript, receive a summary of study findings, or be invited to participate in future related research. Consent may be withdrawn at any time without consequence. Participants will confirm their consent by typing their name and checking a legal agreement box and will receive a PDF copy for their records. Any signed form will be stored securely on the REDCap server.

Data Access and Confidentiality

All data will be de-identified to maintain patient and provider confidentiality. Participant data will be stored on secure servers hosted by the RI-MUHC for up to seven years following the completion of study recruitment. Afterwards, all data will then be destroyed. Any personal identifiers will be separated from the study data with linkage maintained only by the Principal

For peer review only - http://bmjopen.bmj.com/site/about/guidelines.xhtml

Investigator (PI) and study coordinator. If data is transferred to other collaborating institutions, other lead researchers and co-investigators affiliated with the study. Secure servers and formal data transfer agreements will be used to maintain compliance with the confidentiality protocols.

Information collected from participants will remain strictly confidential within the limits provided by the law. Each participant will be assigned an identification number upon recruitment so that their personal information and the information collected as a research subject will be stored in two separate databases. All study data will be collected using REDCap. Participants' contact information and survey responses will be kept in the same project space but only accessible by the PI and study coordinator. REDCap will automatically flag and restrict access to identifiers and remove them from all exports unless explicitly permitted.

Audio/Video Recording and Transcription

The qualitative interviews will be audio and/or video recorded when using a videoconferencing platform (Zoom or Teams) and when conducting the qualitative interviews in-person. In-person qualitative interviews will be videorecorded using a digital camera. Data from the SD card of the camera will be transferred to the research team's secured server housed and once uploaded to the server, it will immediately be deleted from the SD card. Each video recording will be saved using a file name that contains a random number and the date of the recording. A separate, password-protected Excel spreadsheet saved on the study's secure server will link the video's file name with the participant's unique study ID. This Excel spreadsheet will only be accessible to the PI, the research coordinator and the research assistants responsible for carrying out the qualitative interviews. Additionally, only the PI, research coordinator and research assistants responsible for transcription of the videos will have access to the folder where

For peer review only - http://bmjopen.bmj.com/site/about/guidelines.xhtml

the video recordings are saved. Trained research personnel will transcribe the interview via REDCap. The transcription on REDCap will be associated with the participant's unique study ID and will not contain any identifiable information. Video recordings will be destroyed seven years from the date of completion of recruitment for the study.

Dissemination

Knowledge mobilization will be carried out as end-of-grant dissemination activities and will include the preparation and writing of papers, as well as clinical rounds, conference presentations and media op-eds. The knowledge findings derived from this larger research project implementation have the potential to promote parental engagement in the NICU as a key predictor of family resilience and associated child more immediate and long-term well-being. Early life adversity has implications for longer term global functioning into adulthood, thus attesting to the importance and timeliness of this research.

For peer review only - http://bmjopen.bmj.com/site/about/guidelines.xhtml

REFERENCES

1. Gee DG. Neurodevelopmental mechanisms linking early experiences and mental health: Translating science to promote well-being among youth. Am Psychol. 2022;77(9):1033–45.

2. Spittle A, Treyvaud K. The role of early developmental intervention to influence neurobehavioral outcomes of children born preterm. Semin Perinatol. 2016 Dec;40(8):542–8.

3. van Wyk L, Majiza AP, Ely CSE, Singer LT. Psychological distress in the neonatal intensive care unit: a meta-review. Pediatr Res. 2024 Nov;96(6):1510–8.

4. Shaw RJ, Bernard RS, Storfer-Isser A, Rhine W, Horwitz SM. Parental Coping in the Neonatal Intensive Care Unit. J Clin Psychol Med Settings. 2013 Jun;20(2):135–42.

5. Laccetta G, Di Chiara M, De Nardo MC, Terrin G. Symptoms of post-traumatic stress disorder in parents of preterm newborns: A systematic review of interventions and prevention strategies. Front Psychiatry [Internet]. 2023 Mar 8 [cited 2025 Jun 4];14. Available from: https://www.frontiersin.org/journals/psychiatry/articles/10.3389/fpsyt.2023.998995/full

6. Caporali C, Pisoni C, Gasparini L, Ballante E, Zecca M, Orcesi S, et al. A global perspective on parental stress in the neonatal intensive care unit: a meta-analytic study. J Perinatol. 2020 Dec;40(12):1739–52.

7. Feudtner C, Walter JK, Faerber JA, Hill DL, Carroll KW, Mollen CJ, et al. Good-parent beliefs of parents of seriously ill children. JAMA Pediatr. 2015 Jan;169(1):39–47.

8. Greene MM, Rossman B, Patra K, Kratovil AL, Janes JE, Meier PP. Depression, Anxiety, and Perinatal-Specific Posttraumatic Distress in Mothers of Very Low Birth Weight Infants in the Neonatal Intensive Care Unit. J Dev Behav Pediatr. 2015 Jun;36(5):362.

9. Latendresse G, Wong B, Dyer J, Wilson B, Baksh L, Hogue C. Duration of Maternal Stress and Depression: Predictors of Newborn Admission to Neonatal Intensive Care Unit and Postpartum Depression. Nurs Res. 2015 Oct;64(5):331.

10. Vasa R, Eldeirawi K, Kuriakose VG, G JN, Newsom C, Bates J. Postpartum Depression in Mothers of Infants in Neonatal Intensive Care Unit: Risk Factors and Management Strategies. Am J Perinatol. 2013 Aug 5;31:425–34.

11. Beck CT, Watson S, Gable RK. Traumatic Childbirth and Its Aftermath: Is There Anything Positive? J Perinat Educ. 2018 Jun 1;27(3):175–84.

12. Haward MF, Lantos J, Janvier A, for the POST Group. Helping Parents Cope in the NICU. Pediatrics. 2020 Jun 1;145(6):e20193567.

For peer review only - http://bmjopen.bmj.com/site/about/guidelines.xhtml

13. Lean RE, Rogers CE, Paul RA, Gerstein ED. NICU Hospitalization: Long-Term Implications on Parenting and Child Behaviors. Curr Treat Options Pediatr. 2018 Mar;4(1):49–69.

14. Murdoch MR, Franck LS. Gaining confidence and perspective: a phenomenological study of mothers’ lived experiences caring for infants at home after neonatal unit discharge. J Adv Nurs. 2012 Sep;68(9):2008–20.

15. O’Brien K, Robson K, Bracht M, Cruz M, Lui K, Alvaro R, et al. Effectiveness of Family Integrated Care in neonatal intensive care units on infant and parent outcomes: a multicentre, multinational, cluster-randomised controlled trial. Lancet Child Adolesc Health. 2018 Apr;2(4):245–54.

16. Umberger E, Canvasser J, Hall SL. Enhancing NICU parent engagement and empowerment. Semin Pediatr Surg. 2018 Feb;27(1):19–24.

17. Novak JL, Vittner D. Parent engagement in the NICU. J Neonatal Nurs. 2021 Aug 1;27(4):257–62.

18. Boykova M, Kenner C. Transition from hospital to home for parents of preterm infants. J Perinat Neonatal Nurs. 2012;26(1):81–7; quiz 88–9.

19. Holditch-Davis D, Miles MS. Mothers’ stories about their experiences in the neonatal intensive care unit. Neonatal Netw NN. 2000 Apr;19(3):13–21.

20. Association of Women’s Health, Obstetric and Neonatal Nurses. Assessment and Care of the Late Preterm Infant. J Obstet Gynecol Neonatal Nurs JOGNN. 2024 Sep;53(5):e1–62.

21. Albanese AM, Russo GR, Geller PA. The role of parental self-efficacy in parent and child well-being: A systematic review of associated outcomes. Child Care Health Dev. 2019 May;45(3):333–63.

22. Barnes CR, Adamson-Macedo EN. Perceived Maternal Parenting Self-Efficacy (PMP S-E) tool: development and validation with mothers of hospitalized preterm neonates. J Adv Nurs. 2007 Dec;60(5):550–60.

23. Bandura A. Self-efficacy: Toward a unifying theory of behavioral change. Psychol Rev. 1977;84(2):191–215.

24. Pineda R, Bender J, Hall B, Shabosky L, Annecca A, Smith J. Parent Participation in the Neonatal Intensive Care Unit: Predictors and Relationships to Neurobehavior and Developmental Outcomes. Early Hum Dev. 2018 Feb;117:32–8.

25. Silva N de F, Linhares MBM, Gaspardo CM. Stress and self-regulation behaviors in preterm neonates hospitalized at open-bay and single-family room Neonatal Intensive Care Unit. Infant Behav Dev. 2024 Sep 1;76:101951.

For peer review only - http://bmjopen.bmj.com/site/about/guidelines.xhtml

26. Rutherford HJV, Wallace NS, Laurent HK, Mayes LC. Emotion Regulation in Parenthood. Dev Rev DR. 2015 Jun 1;36:1–14.

27. Dell'Aversana V, Tofani M, Valente D. Emotional Regulation Interventions on Developmental Course for Preterm Children: A Systematic Review of Randomized Control Trials. Children. 2023 Mar 22;10(3):603.

28. Hamzallari O, Rosinski L, Petrenko A, Bridgett DJ. Mothers' Emotion Regulation and Negative Affect in Infants: The Role of Self-Efficacy and Knowledge of Parenting Practices. Children. 2022 Dec 31;10(1):85.

29. Siva N, Nayak BS, Edward S Lewis L, Velayudhan B, Noronha JA. Intervention strategies to enhance early mother-infant interaction at NICU - A systematic review. J Neonatal Nurs. 2025 Jun 1;31(3):101671.

30. Almalki M, Gildea A, Boyle B. Parents' experiences of family-centred care in neonatal intensive care units: A qualitative thematic synthesis. J Neonatal Nurs. 2025 Jun 1;31(3):101655.

31. McCarty DB, Ferrari RM, Golden S, Zvara BJ, Wilson WD, Shanahan ME. Identifying Facilitators and Barriers to Neonatal Intensive Care Unit Visitation in Mothers of Low Socioeconomic Status: A Qualitative Investigation. Children. 2024 Nov 16;11(11):1390.

32. Neu M, Klawetter S, Greenfield JC, Roybal K, Scott JL, Hwang SS. Mothers' Experiences in the NICU Before Family-Centered Care and in NICUs Where It Is the Standard of Care. Adv Neonatal Care Off J Natl Assoc Neonatal Nurses. 2020 Feb;20(1):68–79.

33. Almalki M, Gildea A, Boyle B. Parents' experiences of family-centred care in neonatal intensive care units: A qualitative thematic synthesis. J Neonatal Nurs. 2025 Jun 1;31(3):101655.

34. Garrand S, Sherman N, Rentchier D, Jung AL. A Pa rent-to-Parent Program. Fam Community Health. 1978 Nov;1(3):103.

35. Dahan S, Bourque CJ, Reichherzer M, Prince J, Mantha G, Savaria M, et al. Community, Hope, and Resilience: Parental Perspectives on Peer Support in Neonatology. J Pediatr. 2022 Apr 1;243:85-90.e2.

36. Breastfeeding Peer Counselors as Direct Lactation Care Providers in the Neonatal Intensive Care Unit - Paula P. Meier, Janet L. Engstrom, Beverly Rossman, 2013 [Internet]. [cited 2025 Jul 1]. Available from: https://journals.sagepub.com/doi/abs/10.1177/0890334413482184

37. Mangurten HH, Slade C, Fitzsimons D. Parent-Parent Support in the Care of High-Risk Newborns. J Obstet Gynecol Neonatal Nurs. 1979;8(5):275–7.

38. Treyvaud K. Parent and family outcomes following very preterm or very low birth weight birth: A review. Semin Fetal Neonatal Med. 2014 Apr 1;19(2):131–5.

For peer review only - http://bmjopen.bmj.com/site/about/guidelines.xhtml

39. Toral-López I, Fernández-Alcántara M, González-Carrión P, Cruz-Quintana F, Rivas-Campos A, Pérez-Marfil N. Needs Perceived by Parents of Preterm Infants: Integrating Care Into the Early Discharge Process. J Pediatr Nurs Nurs Care Child Fam. 2016 Mar 1;31(2):e99–108.

40. Hearn G, Clarkson G, Day M. The Role of the NICU in Father Involvement, Beliefs, and Confidence: A Follow-up Qualitative Study. Adv Neonatal Care. 2020 Feb;20(1):80.

41. Madigan S, Atkinson L, Laurin K, Benoit D. Attachment and internalizing behavior in early childhood: A meta-analysis. Dev Psychol. 2013;49(4):672–89.

42. Ardila A, Rosselli M, Matute E, Inozemtseva O. Gender differences in cognitive development. Dev Psychol. 2011;47(4):984–90.

43. Garfield CF, Kerrigan E, Christie R, Jackson KL, Lee YS. A Mobile Health Intervention to Support Parenting Self-Efficacy in the Neonatal Intensive Care Unit from Admission to Home. J Pediatr. 2022 May 1;244:92–100.

44. Larocque C, Peterson WE, Squires JE, Mason-Ward M, Mayhew K, Harrison D. Family-centred care in the Neonatal Intensive Care Unit: A concept analysis and literature review. J Neonatal Nurs. 2021 Dec 1;27(6):402–11.

45. Malin KJ, Vittner D, Darilek U, McGlothen-Bell K, Crawford A, Koerner R, et al. Application of the Adverse Childhood Experiences Framework to the NICU. Adv Neonatal Care Off J Natl Assoc Neonatal Nurses. 2024 Feb 1;24(1):4–13.

46. Senechal E, Radeschi D, Kearney R, Shalish W, Sant'Anna G. Parents and Health Care Providers' Perspectives on Vital Signs Monitoring Technologies in the Neonatal Intensive Care Unit: An International Survey. Am J Perinatol. 2025 May 29;

47. Dall'Oglio I, Mascolo R, Portanova A, Ragni A, Amadio P, Fiori M, et al. Staff Perceptions of Family-Centered Care in Italian Neonatal Intensive Care Units: A Multicenter Cross-Sectional Study. Children. 2022 Sep;9(9):1401.

48. Lake ET, Hallowell SG, Kutney-Lee A, Hatfield LA, Del Guidice M, Boxer B, et al. Higher Quality of Care and Patient Safety Associated with Better NICU Work Environments. J Nurs Care Qual. 2016;31(1):24–32.

49. Lou NM, Montreuil T, Feldman LS, Fried GM, Lavoie-Tremblay M, Bhanji F, et al. Evaluations of Healthcare Providers' Perceived Support From Personal, Hospital, and System Resources: Implications for Well-Being and Management in Healthcare in Montreal, Quebec, During COVID-19. Eval Health Prof. 2021 Sep 1;44(3):319–22.

50. Ryynänen SP. Patient Process-Based Well-Being With the Support of a Close Person. J Patient Exp. 2023 Jan 1;10:23743735231155802.

51. Marzban S, Najafi M, Agolli A, Ashrafi E. Impact of Patient Engagement on Healthcare Quality: A Scoping Review. J Patient Exp. 2022 Jan 1;9:23743735221125439.

For peer review only - http://bmjopen.bmj.com/site/about/guidelines.xhtml

52. Rizzolo A, Shah PS, Bertelle V, Makary H, Ye XY, Abenhaim HA, et al. Association of timing of birth with mortality among preterm infants born in Canada. J Perinatol Off J Calif Perinat Assoc. 2021 Nov;41(11):2597–606.

53. Harris PA, Taylor R, Thielke R, Payne J, Gonzalez N, Conde JG. Research electronic data capture (REDCap)—A metadata-driven methodology and workflow process for providing translational research informatics support. J Biomed Inform. 2009 Apr 1;42(2):377–81.

54. Veenendaal NR van, Auxier JN, Schoor SRD van der, Franck LS, Stelwagen MA, Groof F de, et al. Development and psychometric evaluation of the CO-PARTNER tool for collaboration and parent participation in neonatal care. PLOS ONE. 2021 Jun 9;16(6):e0252074.

55. Lake ET, Smith JG, Staiger DO, Schoenauer KM, Rogowski JA. Measuring Parent Satisfaction With Care in Neonatal Intensive Care Units: The EMPATHIC-NICU-USA Questionnaire. Front Pediatr [Internet]. 2020 Oct 6 [cited 2025 Jun 4];8. Available from: https://www.frontiersin.org/journals/pediatrics/articles/10.3389/fped.2020.541573/full

56. Condon JT. Maternal Postnatal Attachment Scale [Measurement Instrument] [Internet]. 2015 [cited 2025 Jun 4]. Available from: https://doi.org/10.25957/5dc0f28d14338

57. Condon JT. Paternal Postnatal Attachment Scale [Measurement instrument] [Internet]. Flinders University. 2015 [cited 2025 Jun 4]. Available from: https://doi.org/10.25957/5dc0f8641433a

58. Zimet GD, Dahlem NW, Zimet SG, Farley GK. The Multidimensional Scale of Perceived Social Support. J Pers Assess. 1988;52(1):30–41.

59. Williams TS, McDonald KP, Roberts SD, Westmacott R, Ahola Kohut S, Dlamini N, et al. In their own words: developing the Parent Experiences Questionnaire following neonatal brain injury using participatory design. Brain Inj. 2018;32(11):1386–96.

60. Johnston C, and Mash EJ. A Measure of Parenting Satisfaction and Efficacy. J Clin Child Psychol. 1989 Jun 1;18(2):167–75.

61. Berry JO, Jones WH. The Parental Stress Scale: Initial Psychometric Evidence. J Soc Pers Relatsh. 1995 Aug 1;12(3):463–72.

62. Cohen S, Kamarck T, Mermelstein R. A Global Measure of Perceived Stress. J Health Soc Behav. 1983;24(4):385–96.

63. Samra HA, McGrath JM, Fischer S, Schumacher B, Dutcher J, Hansen J. The NICU Parent Risk Evaluation and Engagement Model and Instrument (PREEMI) for Neonates in Intensive Care Units. J Obstet Gynecol Neonatal Nurs. 2015 Jan 1;44(1):114–26.

64. Gierk B, Kohlmann S, Kroenke K, Spangenberg L, Zenger M, Brähler E, et al. The somatic symptom scale-8 (SSS-8): a brief measure of somatic symptom burden. JAMA Intern Med. 2014 Mar;174(3):399–407.

For peer review only - http://bmjopen.bmj.com/site/about/guidelines.xhtml

65. Spielberger CD. State-trait anxiety inventory: Bibliography, 2nd edn. Palo Alto. CA: Consulting Psychologists Press; 1989.

66. Lumivero. NVivo Qualitative Data Analysis Software [Internet]. 2017. Available from: www.lumivero.com

67. Guttmann KF, Raviv GN, Fortney CA, Ramirez M, Smith CB. Parent Perspectives on Communication Quality in the Neonatal Intensive Care Unit. Adv Neonatal Care Off J Natl Assoc Neonatal Nurses. 2024 Aug 1;24(4):382–8.

68. Grunberg VA, Presciutti A, Vranceanu AM, Lerou PH. Parental Self-Efficacy and Personal Time Help Explain Impact of Parent-Staff Interactions on Parental Distress and Bonding in the Neonatal Intensive Care Unit. J Pediatr. 2025 Jan;276:114300.

69. Melnyk BM, Feinstein NF, Alpert-Gillis L, Fairbanks E, Crean HF, Sinkin RA, et al. Reducing premature infants' length of stay and improving parents' mental health outcomes with the Creating Opportunities for Parent Empowerment (COPE) neonatal intensive care unit program: a randomized, controlled trial. Pediatrics. 2006 Nov;118(5):e1414-1427.

70. Turner M, Chur-Hansen A, Winefield H, Stanners M. The assessment of parental stress and support in the neonatal intensive care unit using the Parent Stress Scale - Neonatal Intensive Care Unit. Women Birth J Aust Coll Midwives. 2015 Sep;28(3):252–8.

71. Flacking R, Lehtonen L, Thomson G, Axelin A, Ahlqvist S, Moran VH, et al. Closeness and separation in neonatal intensive care. Acta Paediatr Oslo Nor 1992. 2012 Oct;101(10):1032–7.

72. Benzies KM, Magill-Evans JE, Hayden KA, Ballantyne M. Key components of early intervention programs for preterm infants and their parents: a systematic review and meta-analysis. BMC Pregnancy Childbirth. 2013;13 Suppl 1(Suppl 1):S10.

73. Johnson LC, McManus B, Blanchard Y, Nugent JK. The newborn behavioural observations system: A relationship-building intervention to support families in the neonatal intensive care unit. Acta Paediatr. 2025;114(4):752–9.

74. Franck LS, O'Brien K. The evolution of family-centered care: From supporting parent-delivered interventions to a model of family integrated care. Birth Defects Res. 2019 Sep;111(15):1044–59.

75. Government of Canada IAP on RE. Tri-Council Policy Statement: Ethical Conduct for Research Involving Humans – TCPS 2 (2022) [Internet]. 2023 [cited 2025 Jun 4]. Available from: https://ethics.gc.ca/eng/policy-politique_tcps2-eptc2_2022.html

76. WMA - The World Medical Association-WMA Declaration of Helsinki – Ethical Principles for Medical Research Involving Human Participants [Internet]. [cited 2025 Jun 4]. Available from: https://www.wma.net/policies-post/wma-declaration-of-helsinki/

For peer review only - http://bmjopen.bmj.com/site/about/guidelines.xhtml

AUTHOR CONTRIBUTIONS

Concept and design: T.C.M Funding acquisition: T.C.M.; M.B.R.. Drafting of the manuscript: T.C.M., K.M., C.G., M.L.. Critical revision of the manuscript for important intellectual content: T.C.M., K.M., C.G., M.B., M.L.. Manuscript review and editing: All authors meet the full authorship criteria.

Tina C. Montreuil is the Gurantor.

FUNDING STATEMENT

This study protocol was funded via a Canadian Institutes of Health Research-CIHR (2023) Planning and Dissemination Grant (Grant #486178). Funds will support conducting interviews with parents and clinicians to collect data on lived experience, needs, and preference with respect to possible intervention, as well as the current barriers to developing a parental sense of personal self-efficacy, which are critical to the well-being of the developing child and new family. Dr. Tina Montreuil is supported by a Fonds de Recherche du Québec (FRQS) – Research Scholar Salary Junior 2 Award (https://ror.org/01pxwe438). Top up funds were also provided by the Montreal Children Hospital Foundation to support the research project.

CONFLICTS OF INTERESTS STATEMENT

The authors have no competing interests to declare.

ACKNOWLEDGEMENTS

For peer review only - http://bmjopen.bmj.com/site/about/guidelines.xhtml

The authors wish to thank the parents of the Montreal Children's Hospital NICU for their anticipated participation, openness and trust in sharing their experiences. They also wish to acknowledge the NICU staff and healthcare providers for their valuable support and collaboration in the facilitation of the study.

For peer review only - http://bmjopen.bmj.com/site/about/guidelines.xhtml
