# Supplementary material for: Optimising parental self-efficacy in the neonatal intensive care unit (NICU) and its implications on child care and parenting quality: a study protocol
Source: BMJ Open. 2026 May 25;16(5):e116985. doi: 10.1136/bmjopen-2026-116985 (PMC13223963; doi:10.1136/bmjopen-2026-116985)
Supplement: Reviewer comments [file bmjopen-2026-116985.reviewer_comments.pdf]

PEER REVIEW HISTORY

BMJ Open publishes all reviews undertaken for accepted manuscripts. Reviewers are asked to complete a checklist review form and are provided with free text boxes to elaborate on their assessment. These free text comments are reproduced below.

ARTICLE DETAILS

Title (Provisional)

Optimizing Parental Self-Efficacy in the Neonatal Intensive Care Unit (NICU) and its Implications on Child Care and Parenting Quality: A Study Protocol

Authors

Montreuil, Tina; Gratton, Chloé; Mir-Shokraei, Kimia; Biran, Valérie; Beltempo, Marc; Brossard-Racine, Marie; Lecorguillé, Marion

---

VERSION 1 - REVIEW

Reviewer 1

Name Kieran, Emily

Affiliation BC Women's Hospital and Health Centre, Division of Neonatology

Date 12-Apr-2026

COI None

Page 10, line 29: remove 'of'

Page 12, Line 10: Suggest expanding the name or explaining what is a RAMQ # - to help non Canadian readers

---

VERSION 1 - AUTHOR RESPONSE

REVIEWER 1	
Page 10, line 29: remove 'of'	The manuscript has been revised to remove the “of” as instructed by the reviewer. The change is visible on page 8 of the resubmiWed manuscript.

Page 12, Line 10: Suggest expanding the name or explaining what is a RAMQ # - to help non Canadian readers	The RAMQ acronyms has been revised to include the full term. The revised manuscript now reads « Régie de l'assurance maladie du Québec (RAMQ) » and can be found in RED on page 10 of the revised manuscript.
